# ThermomiR-377-3p-induced suppression of Cirbp expression is required for effective elimination of cancer cells and cancer stem-like cells by hyperthermia

**DOI:** 10.1186/s13046-024-02983-3

**Published:** 2024-02-29

**Authors:** Tao-Yan Lin, Jun-Shuang Jia, Wei-Ren Luo, Xiao-Lin Lin, Sheng-Jun Xiao, Jie Yang, Jia-Wei Xia, Chen Zhou, Zhi-Hao Zhou, Shu-Jun Lin, Qi-Wen Li, Zhi-Zhi Yang, Ye Lei, Wen-Qing Yang, Hong-Fen Shen, Shi-Hao Huang, Sheng-Chun Wang, Lin-Bei Chen, Yu-Lin Yang, Shu-Wen Xue, Yong-Long Li, Guan-Qi Dai, Ying Zhou, Ying-Chun Li, Fang Wei, Xiao-Xiang Rong, Xiao-Jun Luo, Bing-Xia Zhao, Wen-Hua Huang, Dong Xiao, Yan Sun

**Affiliations:** 1 Laboratory Animal Management Center, Cancer Research Institute, School of Basic Medical Sciences, Southern Medical University, Guangzhou, 510515 China; 2 Department of Pharmacy, Nanfang Hospital, Southern Medical University, Guangzhou, 510515 China; 3 Medical Research Institute, Guangdong Provincial People’s Hospital (Guangdong Academy of Medical Sciences), Southern Medical University, Guangzhou, 510080 China; 4 Cancer Research Institute, The Second Affiliated Hospital of Southern University of Science and Technology, Shenzhen Third People’s Hospital, Shenzhen, 518112 China; 5 Cancer Center, Integrated Hospital of Traditional Chinese Medicine, Southern Medical University, Guangzhou, 510315 China; 6 Department of Pathology, The Second Affiliated Hospital of Guilin Medical University, Guilin, 541199 China; 7 Department of Imaging, Central Hospital of Shaoyang, Shaoyang, 422000 China; 8 The Third People’s Hospital of Kunming (The Sixth Affiliated Hospital of Dali University), Kunming, 650041 China; 9 School of Laboratory Medicine and Biotechnology, Southern Medical University, Guangzhou, 510515 China; 10 Department of Pathology, School of Basic Medicine, Guangdong Medical University, Dongguan, 523808 China; 11 Department of Oncology, Nanfang Hospital, Southern Medical University, Guang‑zhou, 510515 China; 12 Guangdong Engineering Research Center for Translation of Medical 3D Printing Application, Guangdong Provincial Key Laboratory of Medical Biomechanics, National Key Discipline of Human Anatomy, School of Basic Medical Sciences, Southern Medical University, Guangzhou, 510515 China; 13 Guangdong Medical Innovation Platform for Translation of 3D Printing Application, The Third Affiliated Hospital of Southern Medical University, Southern Medical University, Guangzhou, 510000 China; 14 Orthopaedic Center, Affiliated Hospital of Guangdong Medical University, Guangdong Medical University, Zhanjiang, 524001 China; 15 Guangzhou Southern Medical Laboratory Animal Sci.&Tech. Co.,Ltd, Guangzhou, 510515 China; 16 National Demonstration Center for Experimental Education of Basic Medical Sciences, Southern Medical University, Guangzhou, 510515 China; 17 Department of Stomatology, Guangdong Provincial People’s Hospital (Guangdong Academy of Medical Sciences), Southern Medical University, Guangzhou, 510080 China

**Keywords:** Hyperthermia, Nasopharyngeal carcinoma (NPC), Cold-inducible RNA-binding protein (Cirbp), Temperature-sensitive miRNA-377-3p (termed thermomiR-377-3p), Cancer stem cells (CSCs), Chemotherapy, Radiotherapy, Therapy resistance, DNA damage, Apoptosis, Thermosensitivity, Near-infrared (NIR) laser

## Abstract

**Background:**

In recent years, the development of adjunctive therapeutic hyperthermia for cancer therapy has received considerable attention. However, the mechanisms underlying hyperthermia resistance are still poorly understood. In this study, we investigated the roles of cold‑inducible RNA binding protein (Cirbp) in regulating hyperthermia resistance and underlying mechanisms in nasopharyngeal carcinoma (NPC).

**Methods:**

CCK-8 assay, colony formation assay, tumor sphere formation assay, qRT-PCR, Western blot were employed to examine the effects of hyperthermia (HT), HT + oridonin(Ori) or HT + radiotherapy (RT) on the proliferation and stemness of NPC cells. RNA sequencing was applied to gain differentially expressed genes upon hyperthermia. Gain-of-function and loss-of-function experiments were used to evaluate the effects of RNAi-mediated Cirbp silencing or Cirbp overexpression on the sensitivity or resistance of NPC cells and cancer stem-like cells to hyperthermia by CCK-8 assay, colony formation assay, tumorsphere formation assay and apoptosis assay, and in subcutaneous xenograft animal model. miRNA transient transfection and luciferase reporter assay were used to demonstrate that Cirbp is a direct target of miR-377-3p. The phosphorylation levels of key members in ATM-Chk2 and ATR-Chk1 pathways were detected by Western blot.

**Results:**

Our results firstly revealed that hyperthermia significantly attenuated the stemness of NPC cells, while combination treatment of hyperthermia and oridonin dramatically increased the killing effect on NPC cells and cancer stem cell (CSC)‑like population. Moreover, hyperthermia substantially improved the sensitivity of radiation‑resistant NPC cells and CSC‑like cells to radiotherapy. Hyperthermia noticeably suppressed Cirbp expression in NPC cells and xenograft tumor tissues. Furthermore, Cirbp inhibition remarkably boosted anti‑tumor‑killing activity of hyperthermia against NPC cells and CSC‑like cells, whereas ectopic expression of Cirbp compromised tumor‑killing effect of hyperthermia on these cells, indicating that Cirbp overexpression induces hyperthermia resistance. ThermomiR-377-3p improved the sensitivity of NPC cells and CSC‑like cells to hyperthermia in vitro by directly suppressing Cirbp expression. More importantly, our results displayed the significantly boosted sensitization of tumor xenografts to hyperthermia by Cirbp silencing in vivo, but ectopic expression of Cirbp almost completely counteracted hyperthermia-mediated tumor cell-killing effect against tumor xenografts in vivo. Mechanistically, Cirbp silencing-induced inhibition of DNA damage repair by inactivating ATM-Chk2 and ATR-Chk1 pathways, decrease in stemness and increase in cell death contributed to hyperthermic sensitization; conversely, Cirbp overexpression-induced promotion of DNA damage repair, increase in stemness and decrease in cell apoptosis contributed to hyperthermia resistance.

**Conclusion:**

Taken together, these findings reveal a previously unrecognized role for Cirbp in positively regulating hyperthermia resistance and suggest that thermomiR-377-3p and its target gene Cirbp represent promising targets for therapeutic hyperthermia.

**Supplementary Information:**

The online version contains supplementary material available at 10.1186/s13046-024-02983-3.

## Background

In recent years, the development of therapeutic hyperthermia for cancer therapy has received considerable attention [2, 23, 45, 50]. Therapeutic hyperthermia is a therapeutic procedure that increases the temperature of tumor-loaded tissues to 40–43 °C, while the biological rationale for hyperthermia therapy is based on a direct cell-killing effect at a heat-shock temperature above 41–42 °C [2, 23, 45, 50]. Because of its advantages of non-toxic side effects, no damage to human normal tissues, no damage to their own immunity and so on, thermotherapy has become the fifth approach of cancer therapy after surgery, radiotherapy, chemotherapy and immunotherapy, and plays a more and more important role in multidiscipline treatment for various cancers [2, 23, 45, 50]. In recent years, a large number of in vitro and in vivo experiments and clinical data demonstrate that as an adjunctive therapy, hyperthermia combined with radiotherapy and/or chemotherapy improves clinical outcome in cancer therapy [2, 23, 45, 50]. More importantly, in recent years, the development of nanotechnology-based cancer hyperthermia, including Nano-Photo-Thermal Therapy (NPTT), Nano-Magnetic Hyperthermia (NMH) and Nano-Ultrasound Hyperthermia (NUH)], is a growing field of cancer-targeted nanomedicine due to the potential for targeted and localized elimination of tumor cells, and great breakthroughs in nanoparticle-mediated cancer thermotherapy have been already attained [6, 22, 29, 45, 86, 101, 119].

Although therapeutic hyperthermia is a promising adjunctive treatment for cancer, some obstacles still remain to be addressed [2, 6, 22, 23, 29, 45, 50, 86, 101, 119]. One subject matter among these is that the underlying mechanisms involved in tumor response to thermotherapy are still largely unknown. At present, there are several lines of evidence showing the underlying mechanisms of tumor response to hyperthermia [1, 33, 75, 92]. A previous study showed that inhibition of telomerase activity enhanced hyperthermia-mediated radiosensitization [1]. Hyperthermia sensitized glioma stem-like cells to radiation by pharmacologically inhibiting AKT signaling [75]. CTGF silencing sensitized resistant cells to hyperthermia in vitro and in vivo [33]. Hyperthermia synergized with chemotherapy by inhibiting PARP1-dependent DNA replication arrest [92]. However, great endeavors will still be needed to fully dissect the molecular mechanisms involved in tumor response to hyperthermia, especially involved in hyperthermia resistance, which is critical to manipulate key pathways to greatly improve the clinical efficacy of hyperthermia.

Cold-inducible RNA binding protein (Cirbp, also known as A18 hnRNP or Cirp), a member of cold shock protein family and a stress-inducible protein, is activated by various cellular stresses, such as heat- and cold-treatment, hypoxia and UV-irradiation [10, 59, 61, 71, 73, 137, 138]. Accumulated evidence reveals that Cirbp has been implicated in different physiological and pathological processes, including cell proliferation and differentiation, cell senescence, cell survival and apoptosis, oxidative stress, DNA damage and repair, immune and inflammatory responses, telomere maintenance, circadian rhythm, spermatogenesis, and tumor formation and progression, etc [4, 10, 35, 59, 61, 73, 137, 138]. Furthermore, Cirbp functions as a tumor suppressor in ovarian carcinoma and endometrial carcinoma, whereas Cirbp exerts its pro-tumorigenic roles in pancreatic cancer, breast cancer, colorectal cancer, lung cancer, melanoma, prostate cancer, bladder cancer and skin squamous cell carcinoma [10, 11, 16, 41, 47, 58, 59, 71, 73, 137, 138], indicating that Cirbp has context-dependent tumor-suppressive and oncogenic functions in oncogenesis and cancer progression. Our previous study revealed that the significantly decreased expression of Cirbp was found in the clinical specimens of human nasopharyngeal carcinoma (NPC) [61].

Of note, several lines of evidence has indicated that Cirbp is associated with cell stresses, including heat and cold [14, 68, 82, 87, 105, 130]. Chronic hypoxia-induced Cirbp hypermethylation attenuated hypothermic cardioprotection via down-regulation of ubiquinone biosynthesis [68]. Therapeutic hypothermia protected photoreceptors through activating Cirbp pathway [105]. The down-regulation of Cirbp expression was observed in male germ cells of mice and humans under heat stress condition [14, 82, 87]. Moreover, in prostate cancer cells, heat treatment upregulated heat shock proteins and down-regulated cold shock proteins [i.e., Cirbp and RNA binding motif protein 3 (Rbm3)] [130]. Taken together, the aforementioned findings indicate that Cirbp might play an important role during hyperthermia treatment for cancer therapy. In this context, however, more direct evidence is needed, and the biological mechanisms remain to be understood, for clarification and characterization of the importance of Cirbp in thermotherapy for cancer treatment.

Human NPC is one of the most common malignant tumors in East and Southeast Asia [13, 17, 106]. At present, the standard therapy for patients with locoregionally advanced NPC is radiation therapy combined with chemotherapy [13, 17, 106]. Furthermore, with the widespread use of intensity-modulated radiation therapy and combined radiochemotherapy, locoregional control has improved substantially, whereas distant metastasis and local recurrence are now the main cause for treatment failure in local advanced NPC [13, 17, 106]. Novel and effective adjuvant therapy (e.g., hyperthermia) for NPC is urgently warranted. In the field of NPC, a small amount of clinical trials preliminarily demonstrated that hyperthermia combined with radiation therapy improved progression-free survival and local progression-free survival of NPC patients, although no increase in overall survival was observed [37, 46, 85, 121]. However, in contrast to other solid cancers [2, 6, 22, 23, 29, 45, 50, 86, 101, 119], the development of effective hyperthermia treatment for NPC didn’t receive considerable attention. Therefore, intensive research work will still be required to develop effective hyperthermia treatment for NPC.

Against this background, in this study, we fully investigated the direct cell-killing activity of hyperthermia alone or combined treated with chemotherapy or radiotherapy on cancer cells and stem-like cancer cells of NPC. On the other hand, we clarified the potential functions of Cirbp in thermotherapy for NPC treatment in vitro and in vivo, and the molecular mechanisms underlying thermoresistance and thermosensitization.

## Materials and methods

### Cell culture

Human NPC cell lines (i.e., CNE2, SUNE1, 5-8F and HONE1-EBV cells) were obtained from Prof. Qiao Tao (Chinese University of Hong Kong, Hong Kong, China), Prof. S.-W. Tsao (University of Hong Kong), Prof. Yixin Zeng (Sun Yat-sen University, Guangzhou, China), Prof. Musheng Zeng (Sun Yat-sen University, Guangzhou, China) and Dr. Dengke Li (Cancer Research Institute, Southern Medical University, Guangzhou, China). These NPC cell lines were cultured in RPMI 1640 medium supplemented with 10% fetal bovine serum (FBS) (Biological Industries, VivaCell and Cegrogen Biotech) in a humidified incubator with 5% CO_2_ at 37 °C. HEK293T cells were maintained in DMEM medium supplemented with 10% FBS in a humidified incubator with 5% CO_2_ at 37 °C. All cell lines were authenticated by short tandem repeat (STR) fingerprinting at the GUANGZHOU IGE BIOTECHNOLOGY Co., Ltd. (Guangzhou, China).

### Development of radioresistant subclone CNE2‑8G cells

To obtain radiation-resistant NPC cell line, CNE2 cells were exposed to repeated X-ray irradiation (IR), and after a total dose of 8 Gy in 8 fractions, a radioresistant monoclone CNE2-8G was obtained.

### RNA isolation, reverse transcription and quantitative real‑time PCR (qRT‑PCR)

RNA isolation, reverse transcription and qRT-PCR were well described previously [12, 54, 56, 61, 63, 64, 97, 104, 118, 125, 133]. The primers used in the qRT-PCR assay are listed in Table S1. GAPDH or U6 snRNA was used as an endogenous control. All samples were normalized to internal controls, and fold changes were calculated through relative quantification (2^−△△Ct^).

### Western blot assay

Western blot was performed according to previous publications [54, 56, 62, 64, 117, 118, 125, 133]. The primary antibodies used for Western blot were listed in Table S2. GAPDH was used as a loading control.

### CCK‑8 assay, colony formation assay, EdU assay, tumor sphere formation assay and apoptosis assay

Tumor-killing activity of hyperthermia, oridonin (Ori) and IR against cancer cells were assessed by Cell Counting Kit-8 (CCK-8) assay, colony formation assay and tumor sphere formation assay. Before performing the aforementioned assays, cancer cells were firstly sham-treated and treated with hyperthermia (at 42 °C or 44 °C for 30 min), treated with Ori (concentration: 0, 20, 40, 60, 80, 100, 120, 140 and 160 μM), treated with hyperthermia (42 °C for 30 min) and Ori (20 μM Ori for 30 min) alone or combined, and treated with hyperthermia (42 °C for 30 min) and IR (4 Gy) alone or combined. Subsequently, these treated cells were further employed in the abovementioned assays. CCK-8 assay (Dojindo), colony formation assay, EdU assay and tumor sphere formation assay were performed as previously described [54, 56, 88, 97, 120, 133].

For the CCK-8 assay, the indicated cells were plated in 96-well plates at 1 × 10^3^ per well in a final volume of 200 μl and then cultured for 6 days. For the colony formation assay, cells were counted and plated at 1 × 10^3^ per well in 6-well plates for 14 days. For tumor sphere formation assay, NPC cells (1 × 10^3^/well) were grown in serum-free DMEM-F12 supplemented with 10 μg/L bFGF, 20 μg/L EGF and 2% B27 in ultra-low adhesion plates (Corning). Two weeks later, spheres were counted by an inverted microscope (Nikon Eclipse Ti-U), and images were acquired. For the EdU assay, the proliferating NPC cells were examined using the Cell-Light EdU In Vitro Imaging Kit (RiboBio) according to the manufacturer’s protocol.

For the apoptosis assay, cell apoptosis rate was determined by flow cytometry with the Annexin V-APC/7-AAD Apoptosis Kit (Keygen) according to the manufacturer’s protocol. Cells were collected, stained with Annexin V and PI for 15 min in the dark, and then analyzed by a FACS Caliber flow cytometer (BD Bioscience).

### Percentages of side population cells (SP cells) analyzed by flow cytometry

NPC cells treated with hyperthermia (at 42 °C or 44 °C for 30 min) were digested with 0.25% trypsin, washed twice with calcium/magnesium-free PBS, resuspended in ice-cold RPMI-1640 medium (supplemented with 2% FBS) at a concentration of 1 × 10^6^ cells/mL, and incubated at 37 °C in a 5% CO_2_ incubator for 90 min. Following this, the changes in the percentage of SP cells were analyzed by flow cytometry (BD FACSAria).

### Plasmids, lentivirus production and transduction

The fragment (519 bp) of Cirbp was amplified from pENTER-Cirbp [purchased from Vigene Biosciences Co., Ltd. (Jinan, China)], and then directly inserted into *Eco*R I and *Bam*H I sites of the lentivirus vector of pCDH-EF1-MCS-GFP-Puro (pLV-con as empty vector) [Cat. # CD550A-1; purchased from System Biosciences (SBI)] to generate pCDH-EF1-Cirbp-GFP-Puro (i.e., pLV-Cirbp). All lentivirus-mediated RNAi knockdown plasmids of Cirbp [purchased from Vigene Biosciences Co., Ltd. (Jinan, China)] were constructed in a modified pLKO.1-puro vector. The shRNA sequences against Cirbp were presented in Table S1.

For miR-377 overexpression, a fragment containing the precursor sequence of human miR-377 was cloned into the lentivirus vector of pEZX-MR02, designated pLV-miR-377 [purchased from GeneCopoeia, Inc. (Guangzhou, China)].

The lentiviral packaging plasmids psPAX2 and pMD2.G were kindly provided by Prof. Didier Trono (University of Geneva, Geneva, Switzerland). To generate stable cell lines, recombinant lentiviruses [named as LV-shSCR (SCR: scrambled control shRNA) and LV-con (used as control), and LV-shCirbp, LV-Cirbp and LV-miR-377] were generated as previously described [54, 88, 97, 133], and subsequently used to infect the indicated cells (i.e., CNE2, SUNE1 and HONE1-EBV cells) to generate shSCR-, vector-, shCirbp-, Cirbp- or miR-377-expressing cancer cell lines, respectively.

### miRNA transient transfection

Mimics-NC, mimics, inhibitors-NC and inhibitors of miR-377-3p and miR-381-3p were purchased from RiboBio (Guangzhou, China). Transient transfection was carried out using Lipofectamine 2000 (Thermo Fisher Scientific) according to the manufacturer’s recommendation.

### Luciferase reporter assay

The dual luciferase reporter gene plasmid (i.e., pLuc-Cirbp-3′-UTR-WT) containing the putative miR-377 binding site at the 3′-UTR of Cirbp mRNA and the corresponding pLuc-Cirbp-3′-UTR-MUT were purchased from Kangbio(Shenzhen, China). Cells were seeded in 48-well plates and cultured for 48 h. The pLuc-Cirbp-3′-UTR-WT or pLuc-Cirbp-3′-UTR-MUT was co-transfected into HEK293T cells with the miR-377-3p mimics, mimics control, miR-377-3p inhibitor or inhibitor control using Lipofectamine 2000 Reagent (Invitrogen), respectively. Luciferase and Renilla activities were assayed 48 h after transfection using the Dual Luciferase Reporter Assay Kit (Promega) following the manufacturer’s instructions.

### Immunofluorescent (IF) staining

IF staining was performed according to the protocol of a standard method described previously [56, 96]. NPC cells grown on coverslips were rinsed with PBS, and then fixed with cold 4% paraformaldehyde for 5 min at room temperature. Subsequently, the cells were permeabilized with 0.3% Triton X-100 for 30 min, and then incubated with primary monoclonal antibodies 53BP1 (Cat. No. ab175933, 1:250, Abcam) and γ-H2AX (Cat. No. ab26350, 1:500, Abcam) for 2 h at room temperature, respectively. After three washes in PBS for 5 min each, the slides were incubated for 1 h in the dark room with goat anti-rabbit IgG (H + L) Dylight 549 and goat anti-Mouse IgG (H + L) Dylight 549 (1:1000, Bioworld Technology, Inc.), respectively. Finally, the slides were counterstained with DAPI (Sigma) for 5 min to visualize the nuclei and imaged with a confocal laser-scanning microscope (Nikon A1). The primary antibodies used for IF staining were listed in Table S2.

### RNA sequencing

Total RNAs were extracted from Cirbp-expressing and shCirbp-expressing NPC cells treated with or without hyperthermia at 42 °C for 30 min using TRIzol Reagent (TaKaRa) following the methods by Chomczynski et al. [15]. DNA digestion was carried out after RNA extraction by DNaseI. RNA quality was determined by examining A260/A280 with Nanodrop™ One spectrophotometer (Thermo Fisher Scientific Inc). RNA integrity was confirmed by 1.5% agarose gel electrophoresis. Qualified RNAs were finally quantified by Qubit3.0 with Qubit™ RNA Broad Range Assay kit (Life Technologies, Q10210). 2 μg total RNAs were used for stranded RNA sequencing library preparation using KC-Digital™ Stranded mRNA Library Prep Kit for Illumina (Cat. No. DR08502, Wuhan Seqhealth Co., Ltd.) following the manufacturer’s instruction. The kit eliminates duplication bias in PCR and sequencing steps, by using unique molecular identifier (UMI) of 8 random bases to label the pre-amplified cDNA molecules. The library products corresponding to 200–500 bps were enriched, quantified and finally sequenced on Novaseq 6000 sequencer (Illumina) with PE150 model.

### Animal procedures and treatments

The animal experiments were carried out in strict accordance with the recommendations in the Guide for the Care and Use of Laboratory Animals of the Southern Medical University. The animal protocol was approved by the Committee on Ethics of Animal Experiments of the Southern Medical University. All surgery was performed under sodium pentobarbital anesthesia, and all efforts were made to minimize suffering of animals.

BALB/c nude mice aged 4 to 5 weeks were purchased from Laboratory Animal Management Center, Southern Medical University (Guangzhou, China) and the Medical Laboratory Animal Center of Guangdong Province (Guangzhou, China). shSCR- and shCirbp-expressing NPC cells (2 × 10^6^), or vector- and Cirbp-expressing NPC cells (2 × 10^6^) were subcutaneously injected into the hind limb of each nude mouse, respectively. Tumors were allowed to grow until they reached 6 to 9 mm in maximal diameter, at which time the mice were randomly divided into control group (LV-shSCR and LV-shCirbp) and treatment group (LV-shSCR and LV-shCirbp, ICG-NIR therapy; ICG: indocyanine green; NIR: near-infrared), and into control group (LV-con and LV-Cirbp) and treatment group (LV-con and LV-Cirbp, ICG-NIR therapy). Subsequently, in treatment groups, 30 min prior to laser irradiation, a single dose of 4 mg/kg of sterile ICG solution was infused by tail vein injection into each nude mouse with tumor burden, followed by a NIR laser treatment (808 nm, 1w/cm^2^) for 10 min. NIR laser system (Shanghai Xilong Optoelectronics Technology Co. Ltd., China) emitting 808 nm light was used in this study [36]. The laser beam diameter is 8 mm, the pulse time is 3 ms and the laser radiant exposure was 2 w/cm^2^. In treatment group, nude mice with tumor burdens were treated by NIR laser irradiation (at 41 °C ~ 43 °C) for 10 min every 2 days. No treatment was applied to the mice in two control groups. A single dose of 4 mg/kg of ICG solution was injected into tail vein of each mouse in study groups. Tumor size was measured with a Vernier caliper every 2 days. Tumor volumes were calculated using the formula a^2^*b/2, where a and b are the shorter and longer diameters of the tumor, respectively. 30 days after cancer cell implantation, mice were sacrificed, and tumor xenografts were dissected, weighed and fixed overnight in 4% paraformaldehyde, dehydrated, paraffin-embedded, sectioned, followed by H&E staining.

### Statistical analysis

Data were presented as mean ± SD or mean ± SEM. Statistical analysis was performed using the SPSS 20.0 software package and Graphpad 5.0 software. Two-tailed Student’s t test was used for comparisons of two independent groups. The One-way ANOVA was used for compare comparisons of multiple groups. Values were statistically significant at *P < 0.05, **P < 0.01 and ^#^*P* < 0.001. NS: not significant.

## Results

### Hyperthermia dramatically attenuated the stemness of NPC cells

**Fig. 1:**
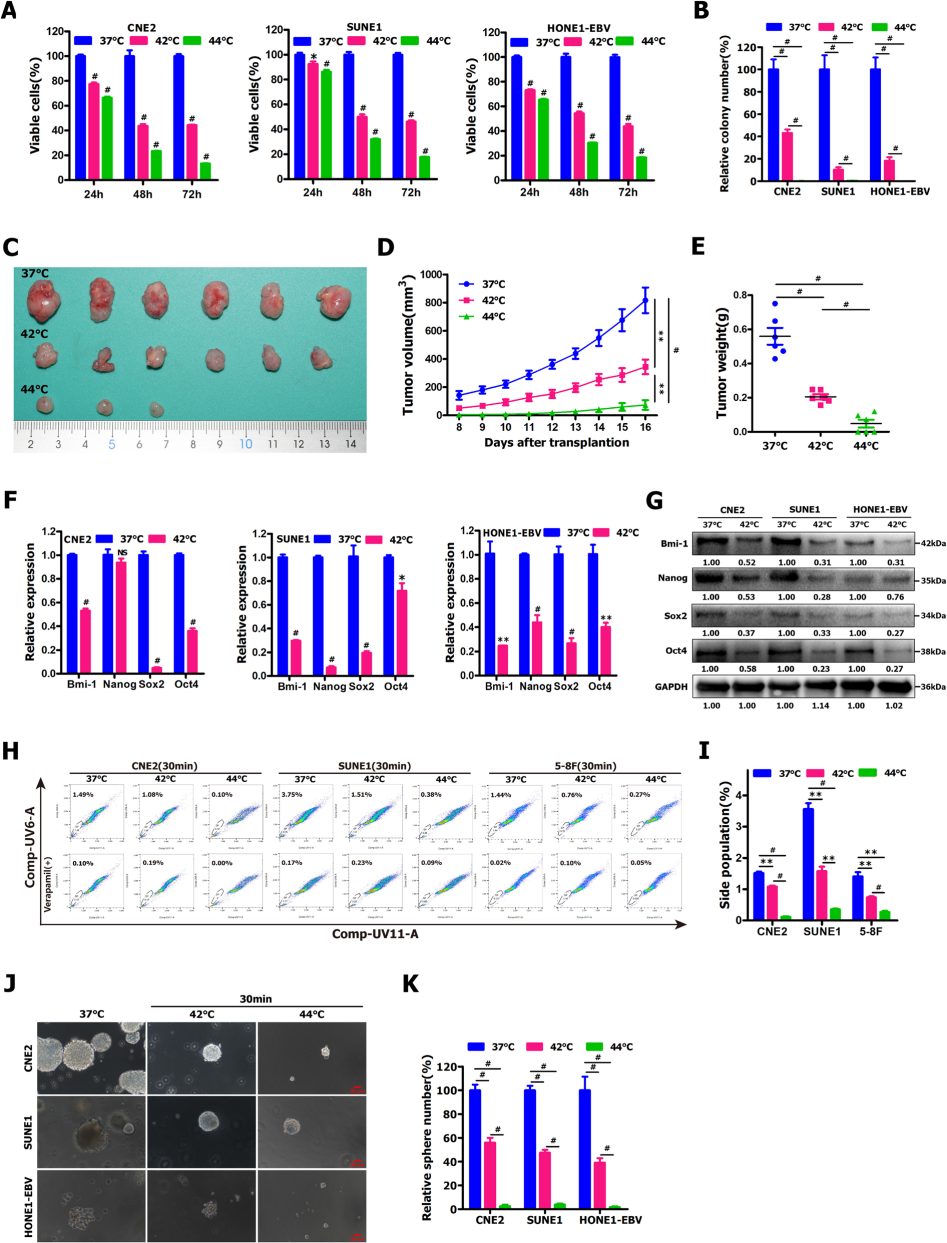
Hyperthermia significantly suppressed the proliferation and stemness of NPC cells. **A**, **B** CCK-8 assay **A** and colony formation assay **B** were performed in indicated NPC cells treated with hyperthermia at 42 °C or 44 °C for 30 min. **C-E** The xenograft subcutaneous tumor formation of hyperthermia-treated CNE2 cells in nude mice. CNE2 cells in vitro treated with hyperthermia at 42 °C or 44 °C for 30 min were injected subcutaneously into nude mice (*n* = 6). **C** Representative images of stripped xenograft tumors formed by CNE2 cells at the end of experiment. **D** The growth curve of tumor volumes within 20 days. **E** Tumor weight. **F**, **G** qRT-PCR **F** and Western blot **G** were employed to detect stemness-related gene expression in indicated NPC cells treated with hyperthermia at 42 °C for 30 min. **H**, **I** Flow cytometry analysis of the percentages of side population (SP) cells in indicated NPC cells treated with hyperthermia at 42 °C or 44 °C for 30 min. **J**, **K** Tumor sphere formation assay was used to detect the self-renewal ability of NPC cells treated with hyperthermia at 42 °C or 44 °C for 30 min. For tumor sphere formation assay, the indicated NPC cells (1 × 10^3^/well) treated with hyperthermia at 42 °C or 44 °C for 30 min were grown in serum-free DMEM-F12 supplemented with 10 μg/L bFGF, 20 μg/L EGF and 2% B27 in ultra-low adhesion plates. Two weeks later, spheres were counted by an inverted microscope, and images were acquired. Sphere size and density are shown in the left panels **J**, and the number of spheres is shown in the right panels **K**

Hyperthermia is the use of elevated temperature for cancer treatment, in this case, typically using temperatures in the range of 41 °C to 45 °C. However, the suitable heat-treating temperature and time for the treatment of NPC cells is unclear. Firstly, we wanted to determine the suitable heat-treating temperature and time for the treatment of NPC cells, and the sensitivity of NPC cells (i.e., CNE2, SUNE1 and HONE1-EBV) to hyperthermia. Colony formation assay showed that the suitable heat-treating temperature and time for NPC cell treatment are 42 °C and 30 min, respectively (Figs. S1 and S2). To examine the anti-tumor activity of hyperthermia treatment, we firstly evaluated the ability of hyperthermia to kill NPC cells (i.e., CNE2, SUNE1 and HONE1-EBV cells) in vitro by using CCK-8 assay. As expected, CCK-8 assay demonstrated that indicated NPC cells treated with hyperthermia at 42 °C or 44 °C for 30 min displayed dramatically decreased cell viability, compared to control group (at 37 °C) (Fig. 1A). Colony formation assay illustrated that heat-treated CNE2, SUNE1 and HONE1-EBV cells (at 42 °C or 44 °C) demonstrated a significantly decreased colony forming ability (Fig. 1B and Fig. S2), compared to no hyperthermia treatment (at 37 °C), while NPC cells treated with hyperthermia at 44 °C exhibited a notably reduced colony forming ability (Fig. 1B and Fig. S2), compared with hyperthermia treatment (at 42 °C). In addition, CNE2 cells in vitro treated with hyperthermia at 42 °C or 44 °C for 30 min displayed a significantly decreased ability of subcutaneous tumor formation in nude mice (Fig. 1C, D, E and Fig. S3). Summarily, these data suggest that thermotherapy has a strong cancer cell killing activity (CKA) in vitro against NPC cells.

Since our results demonstrated that hyperthermia showed a strong CKA in vitro against NPC cells (Fig. 1A, B and Fig. S2), we further explored the effects of thermotherapy treatment on stem cell-like populations within NPC cells by detecting stemness markers, SP cell (side population cell) detecting assay and tumorsphere formation assay. Heat treatment (at 42 °C) in the indicated NPC cells resulted in the remarkable downregulation of stem cell-related markers (i.e., Bmi-1, Nanog, Sox2 and Oct4) at mRNA (Fig. 1F) and protein (Fig. 1G) levels. Furthermore, SP cells within NPC cells and tumorspheres have been reported to exhibit the characteristics of cancer stem cells (CSCs) [24, 49, 56, 70, 116, 120]. We examined the effects of hyperthermia treatment on the percentages of SP cells within NPC cells, and observed that hyperthermia dramatically decreased the percentages of SP cells in CNE2[1.08% (at 42 °C) or 0.11% (at 44 °C) vs. 1.51% (at 37 °C)], SUNE1 [1.57% (at 42 °C) or 0.35% (at 44 °C) vs. 3.56% (at 37 °C)] and 5-8F cells [0.74% (at 42 °C) or 0.27% (at 44 °C) vs. 1.41% (at 37 °C)] (Fig. 1H, I). This data was confirmed on several occasions, and found to be statistically significant (Fig. 1I). Subsequently, we further examined the ability of heat-treated NPC cells to form tumor spheres. Tumorsphere formation assay indicated that hyperthermia-treated NPC cells (at 42 °C or 44 °C) demonstrated a dramatic decrease in tumorsphere formation efficiency (Fig. 1J, K). Together, our results indicate that hyperthermia treatment can efficiently kill cancer stem-like cell populations within NPC cells.

### Combination treatment of hyperthermia and oridonin significantly increased the killing effects on NPC cells and stem‑like cancer cells

**Fig. 2:**
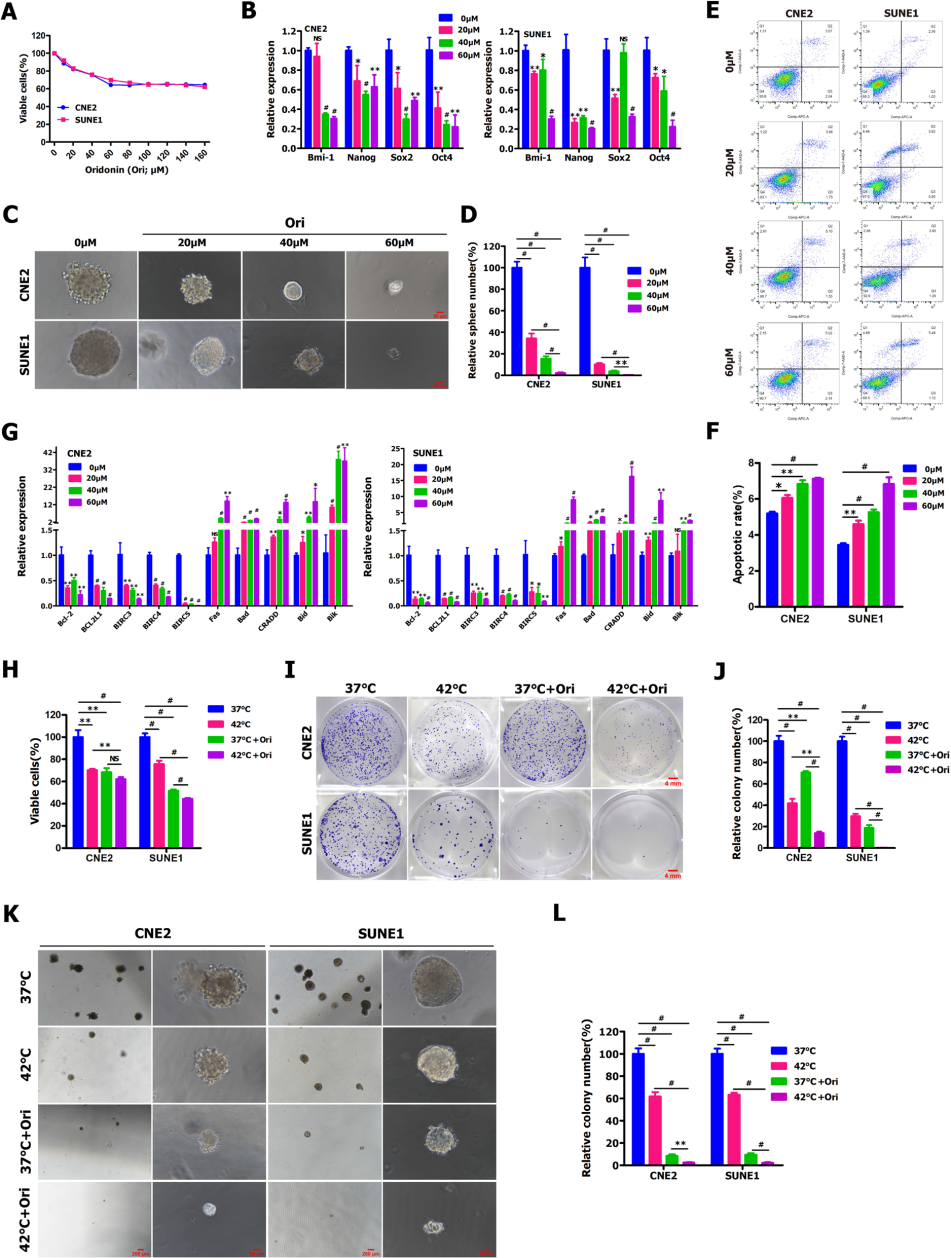
Combination treatment of hyperthermia and oridonin (Ori) significantly increased the killing effects on NPC cells and CSC-like population within NPC cells in vitro. **A** CCK-8 assay was performed in CNE2 and SUNE1 cells treated with oridonin at different concentrations for 24 h. **B** qRT-PCR was used to detect stemness-related gene expression in CNE2 and SUNE1 cells treated with different concentrations of oridonin (20, 40 and 60 μM) for 24 h.** C**, **D** Tumor sphere formation assay was performed in CNE2 and SUNE1 cells treated with oridonin at 20, 40 and 60 μM concentration for 24 h. **E**, **F** AnnexinV/PI apoptosis assay was performed in CNE2 and SUNE1 cells treated with the indicated concentrations of oridonin for 24 h. **G** qRT-PCR was used to detect apoptosis-related gene expression in CNE2 and SUNE1 cells treated with the indicated concentrations of oridonin for 24 h. **H–L** CCK-8 assay **H**, colony formation assay **I**, **J** and tumor sphere formation assay **K**, **L** were performed in CNE2 and SUNE1 cells treated with oridonin (20 μM) alone or combined treated with hyperthermia (42 °C for 30 min)

Natural product oridonin (Ori) and its analogue alone or combined with chemotherapy and radiotherapy were reported to effectively kill tumor cells of leukemia, ovarian cancer, lung cancer, esophageal squamous cell carcinoma, osteosarcoma, breast cancer, colorectal cancer and prostate cancer [38, 55, 72, 74, 78, 84, 94, 102, 112, 122, 126–128, 134, 136]. To explore whether combination treatment of hyperthermia and Ori could significantly enhance the cell-killing effects on NPC cells and stem-like cancer cells, we first evaluated the effects of the different concentrations of Ori on cell viability. As shown in Fig. 2A, the results from CCK-8 assay revealed that high concentrations of Ori markedly reduced cell viability of CNE2 and SUNE1 cells. Subsequently, we further examined the effects of Ori treatment on the stemness maintenance ability of NPC cells by detecting stemness genes and tumorsphere formation assay. qRT-PCR showed that the expression levels of Nanog, Sox2 and Oct4, but not Bmi-1, were dramatically reduced in CNE2 cells upon treatment with 20 μM Ori, while Ori treatment (20 μM) resulted in the significantly decreased expression of Bmi-1, Nanog, Sox2 and Oct4 in SUNE1 cells (Fig. 2B). More importantly, we found that Ori treatment (40 and 60 μM) in CNE2 and SUNE1 cells led to the remarkably reduced expression of stem cell-related markers (i.e., Bmi-1, Nanog, Sox2 or Oct4) (Fig. 2B). Sphere-forming assay illustrated that Ori-treated CNE2 and SUNE1 cells demonstrated a dramatic decrease in tumorsphere formation efficiency in a dose-dependent manner (Fig. 2C, D). Collectively, this work reveals that Ori treatment effectively eliminates CSC-like population within NPC cells.

Since Ori dramatically reduced cell viability and stemness of NPC cells, we want to further determine whether Ori treatment activates the intrinsic apoptotic pathway by AnnexinV/PI apoptosis assay and detecting apoptosis-related gene expression. High concentrations of Ori had pronounced apoptosis-promoting effects on CNE2 and SUNE1 cells (Fig. 2E, F). Additionally, qRT-PCR revealed that high concentrations of Ori led to the markedly downregulated expression of anti-apoptosis genes (i.e., Bcl-2, BCL2L1, BIRC3, BIRC4 and BIRC5) and the significantly upregulated expression of proapoptotic genes (i.e., Fas, Bad, CRADD, Bid and Bik) in CNE2 cells and SUNE1 cells (Fig. 2G). Together, these findings clearly illustrate that the intrinsic apoptotic pathway is really activated by Ori treatment.

Next, we further determined whether combination treatment of hyperthermia and Ori significantly enhanced the antitumor cell-killing effects on NPC cells and stem-like cancer cells by CCK-8 assay, colony formation assay and tumorsphere formation assay. Our results showed that combination treatment of hyperthermia and Ori in CNE2 and SUNE1 cells significantly reduced cell viability (Fig. 2H), colony forming ability (Fig. 2I, J) and tumorsphere formation ability (Fig. 2K, L), compared with hyperthermia or Ori alone. Collectively, the combination treatment of hyperthermia and Ori dramatically increases the antitumor cell-killing effect against NPC cells and CSC-like population.

### Development of radiation‑resistant NPC cell line CNE2‑8G

**Fig. 3:**
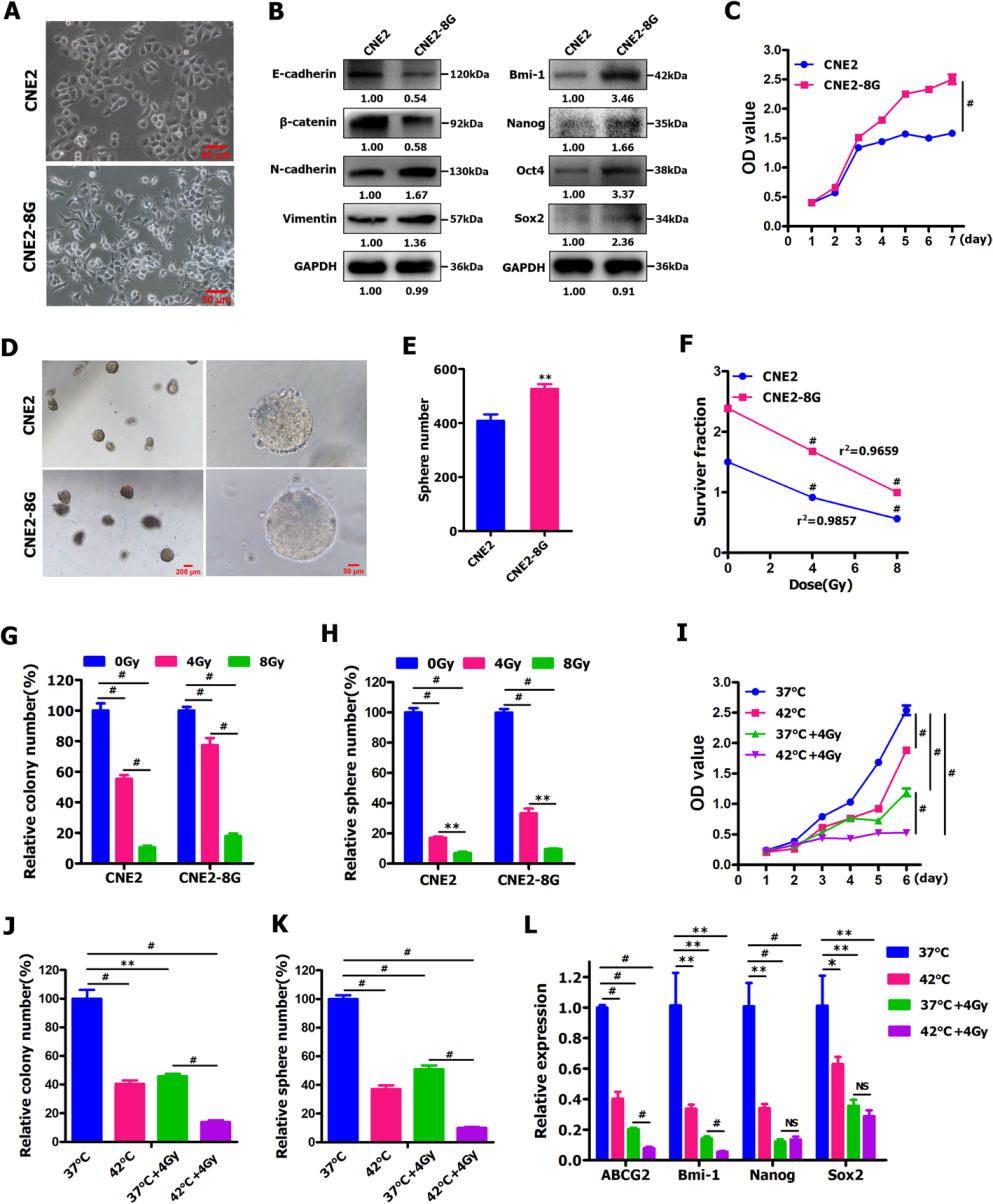
Hyperthermia significantly enhanced the anti-tumor-killing activity of radiotherapy against radiation-resistant NPC cells and cancer stem-like cells. **A** Representative photographs of the morphology of CNE2 cells and radiation-resistant CNE2-8G cells. **B** Western blot was employed to detect stemness- and EMT-related gene expression in CNE2 and CNE2-8G cells. **C** CCK-8 assay was performed in CNE2 and CNE2-8G cells. **D**, **E** Tumor sphere formation assay was performed in CNE2 and CNE2-8G cells. **F–H** CCK-8 assay **F**, colony formation assay **G** and tumor sphere formation assay **H** were performed in CNE2 and CNE2-8G cells subjected to irradiation (IR) treatment at 0, 4 and 8Gy. **I-K** CCK-8 assay **I** colony formation assay **J** and tumor sphere formation assay **K** were performed in CNE2-8G cells treated by hyperthermia (42 °C for 30 min) and IR (4Gy) alone or combined. **L** qRT-PCR assay for detecting stemness-related gene expression in CNE2-8G cells treated by hyperthermia (42 °C for 30 min) and IR (4Gy) alone or combined

The resistance of cancer cells to radiotherapy (RT) is a major obstacle in the clinical treatment of head and neck cancer (HNC), including NPC. As RT is regarded as the mainstay treatment for patients with NPC, we intended to determine whether hyperthermia could significantly boost the anti-tumor effects of RT against radiation-resistant NPC cells and cancer stem-like cells. To that end, we firstly developed the radioresistant NPC cell line according to the experimental procedure described in the section of Materials and Methods. After CNE2 cells were exposed to repeated X-ray IR with a total dose of 8 Gy in 8 fractions, we observed that IR-treated CNE2 cells (designated CNE2-8G cells) underwent the morphological transition from a cuboidal epithelial-like to an elongated mesenchymal-like phenotype (Fig. 3A), indicating the induction of epithelial-mesenchymal transition (EMT) in CNE2-8G cells. This prompted us to characterize the existence of EMT in CNE2-8G cells at the molecular level. Western blot analysis illustrated that spindle-like and fibroblastic morphological conversion was accompanied by the increased expression of mesenchymal markers (i.e., vimentin and N-cadherin) and the reduced expression of epithelial markers (i.e., E-cadherin and β-catenin) in CNE2-8G cells (Fig. 3B). Therefore, these results suggest that CNE2-8G cells display mesenchymal-like morphological change and EMT-like cellular marker alterations.

The previous studies showed that cancer cells (including NPC cells) that undergo EMT have been shown to acquire increased stemness [7, 29, 49, 52, 76, 77, 81]. In this study, we found that CNE2-8G cells undergoing EMT-like phenotype changes displayed the upregulated expression of stem cell-related genes (i.e., Bmi-1, Nanog, Oct4 and Sox2) at protein levels (Fig. 3B), a significantly elevated cell viability (Fig. 3C) and a dramatic increase in tumorsphere formation ability (Fig. 3D, E), compared with control cells (i.e., parental CNE2 cells), suggesting that CNE2-8G cells exhibit an increased stemness. Moreover, CCK-8 assay indicated that compared with CNE2 cells, CNE2-8G cells displayed an obviously elevated cell proliferation capacity even in the absence of IR (Fig. 3C).

It is well known that CSCs have been identified as the main center of therapeutic resistance of cancer cells to conventional radiotherapy, chemotherapy, immunotherapy and thermal therapy [3, 7, 8, 25, 29, 43, 52, 76, 77]. Additionally, IR is known to induce CSC properties [52]. Considering the significantly enhanced CSC-like populations within CNE2-8G cells, we further determined the resistance of CNE2-8G cells to IR by CCK-8 assay (Fig. 3F), colony formation assay (Fig. 3G) and tumorsphere formation assay (Fig. 3H). The survival curves of CNE2-8G cells and CNE2 cells detected by CCK-8 assay suggested that the viability of CNE2-8G cells was significantly higher than that of CNE2 cells before and after IR (4 and 8 Gy) (Fig. 3F), indicating that the established CNE2-8G subclone cells are more radiation-resistant than the parental CNE2 cells. Moreover, IR with 4 Gy caused notably reduced colony formation of CNE2-8G cells by about 22.49% (Fig. 3G and Fig. S4A) and tumorsphere formation of CNE2-8G cells by about 66.75% (Fig. 3H and Fig. S4B), as compared with CNE2-8G cells treated without IR. In contrast, IR with 4 Gy noticeably attenuated colony formation of CNE2 cells by about 44.72% (Fig. 3G and Fig. S4A) and tumorsphere formation of CNE2 cells by about 82.90% (Fig. 3H and Fig. S4B), as compared with CNE2 cells treated without IR. Furthermore, IR with 8 Gy led to substantially decreased colony formation of CNE2-8G cells and CNE2 cells by about 82.06 and 89.34% (Fig. 3G and Fig. S4A), respectively, and resulted in appreciably reduced tumorsphere formation ability of CNE2-8G cells and CNE2 cells by about 90.34 and 92.99% (Fig. 3H and Fig. S4B), respectively, as compared with CNE2-8G cells or CNE2 cells treated without IR. Therefore, the established NPC cell line CNE2-8G shows higher radioresistance than parental cell line CNE2.

### Hyperthermia significantly improved the sensitivity of radiation‑resistant NPC cells and cancer stem‑like cells to radiotherapy

Subsequently, we examined the ability of thermotherapy to improve the radiosensitivity of radiation-resistant CNE2-8G cells by CCK-8 assay (Fig. 3I), colony formation assay (Fig. 3J) and tumorsphere formation assay (Fig. 3K), and detecting stem cell-related gene expression (Fig. 3L). As shown in Fig. 3I, CCK-8 assay suggested that treatment of CNE2-8G cells with combined hyperthermia and IR significantly decreased cell number compared to IR alone, and we found that thermoradiotherapy treatment showed an additive effect in reducing cell viability compared to IR alone. IR alone had statistically significant effect on colony formation (Fig. 3J and Fig. S4C) and tumorsphere formation (Fig. 3K and Fig. S4D) of CNE2-8G cells, as compared with control cells (at 37 °C). Hyperthermia alone appreciably attenuated colony formation by about 59.55% (Fig. 3J and Fig. S4C) and tumorsphere formation by about 62.96% (Fig. 3K and Fig. S4D), as compared with control cells (at 37 °C). The most effective treatment is thermoradiotherapy, which substantially reduced colony formation by about 86.15% (Fig. 3J and Fig. S4C) and tumorsphere formation by about 89.98% (Fig. 3K and Fig. S4D), as compared with control cells (at 37 °C). Furthermore, qRT-PCR assay revealed that thermoradiotherapy led to a dramatically decreased expression of stem cell-related genes (i.e., ABCG2 and Bmi-1) (Fig. 3L), as compared to IR or hyperthermia alone. Together, these abovementioned studies illustrate that hyperthermia significantly improves the sensitivity of radiation-resistant NPC cells and cancer stem-like cells to IR, while thermoradiotherapy is more effective than IR alone in substantially boosting the anti-tumor activity against NPC cells and cancer stem-like cells.

### Hyperthermia significantly suppressed Cirbp expression in NPC cells

Next, we intend to gain insight into the underlying mechanisms via which hyperthermia exerts its tumor-killing effect on NPC cells. It is well known that the cold shock proteins (CSPs), namely Cirbp and Rbm3 are induced upon hypothermia and other forms of cellular stress such as UV radiation and hypoxia [59, 69, 73, 130, 137]. Cirbp expression was down-regulated at elevated temperature in male germ cells of mice and humans [74, 82]. The previous study indicated that Rbm3 expression was reduced during fever/pyrexia, and reduced Rbm3 expression in turn led to elevated expression of Rbm3-targeted temperature-sensitive miRNAs (termed thermomiRs), such as miR-142-5p and miR-143 [123]. Moreover, cytoprotective Rbm3 expression was induced by cooling but suppressed by pyrexia in cardiomyocytes [108]. Together, these aforementioned findings strongly suggest that Cirbp and Rbm3 might be implicated in hyperthermia for cancer therapy.

**Fig. 4:**
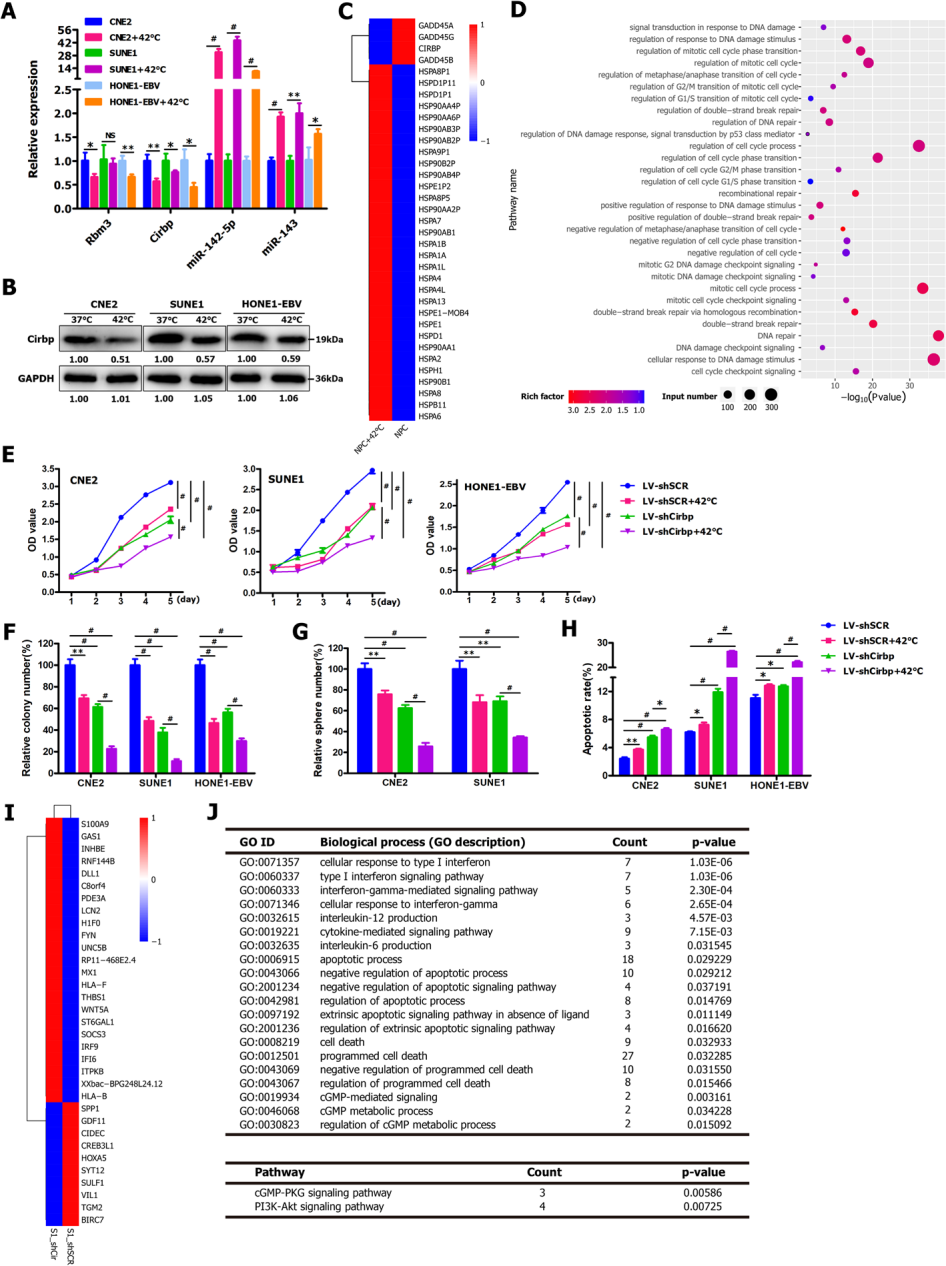
RNAi-mediated silencing of endogenous Cirbp remarkably enhanced the tumor-killing effect of hyperthermia on NPC cells and cancer stem-like cells in vitro. **A** qRT-PCR assay for detecting the expression of Cirbp, Rbm3, miR-143 and miR-142-5p in the indicated NPC cells treated with or without hyperthermia at 42 °C for 30 min. **B** Western blot was employed to detect Cirbp expression in CNE2, SUNE1 and HONE1-EBV cells treated with or without hyperthermia at 42 °C for 30 min. **C** Class comparison and hierarchical clustering of differentially expressed hyperthermia-related genes between NPC cells treated with or without hyperthermia at 42 °C for 30 min. A cluster heat map for upregulated (red) and downregulated (blue) genes (see Tables S3 and S4) is shown. Other details as in Fig. S5. **D** Gene ontology (GO) analysis of up- and down-regulated genes (see Table S3) enriched in hyperthermia-associated biological processes, such as cellular response to heat, DNA damage and repair, cell cycle and cell death between NPC cells treated with or without hyperthermia at 42 °C for 30 min. **E–H** CCK-8 assay **E**, colony formation assay **F**, tumor sphere formation assay **G** and AnnexinV/PI apoptosis assay **H** were performed in shSCR- or shCirbp-expressing NPC cells treated with or without hyperthermia at 42 °C for 30 min. SCR: scrambled control shRNA. **I** Heatmap showing selected differentially expressed genes (see Table S6) related to cell death in shCirbp-expressing NPC cells. Right column lists the selected gene symbols. **J** GO and KEGG pathway analysis of up- and down-regulated genes (see Tables S6 and S7) related to cell survival and death in shSCR and shCirbp-expressing NPC cells

Considering the potentially important roles of Cirbp and Rbm3 in hyperthermia for cancer therapy, we first examined the effects of hyperthermia treatment on the expression of thermomiRs (i.e., miR-142-5p and miR-143) (used as positive control) [123], and Cirbp and Rbm3 by qRT-PCR. The previous study indicated that some miRNAs (i.e., miR-142-5p and miR-143) were identified to belong to temperature-sensitive miRNAs (termed thermomiRs), while fever/pyrexia led to elevated expression of miR-142-5p and miR-143 [123]. NPC cells (i.e., CNE2, SUNE1 and HONE1-EBV) were treated by heating at 42 °C for 30 min. As shown in Fig. 4A, the results from qRT-PCR assay revealed that miR-142-3p and miR-143 were significantly upregulated, and Cirbp was remarkably downregulated in hyperthermia-treated NPC cells (i.e., CNE2, SUNE1 and HONE1-EBV). In contrast, there was no consistent change in Rbm3 expression in three hyperthermia-treated NPC cells (Fig. 4A). In keeping with these aforementioned findings (Fig. 4A), Western blot analysis also showed the dramatically downregulated protein expression of Cirbp in heating-treated CNE2, SUNE1 and HONE1-EBV cells (Fig. 4B).

Furthermore, we performed RNA-sequencing (RNA-seq) in NPC cells treated with or without hyperthermia at 42 °C for 30 min. Comparing hyperthermia-treated NPC cells to control, a total of 9857 differentially expressed genes were identified (Fig. 4C, Fig. S5, Tables S3 and S4) and classified using Gene Ontology (GO) categories (Fig. 4D and Table S5). GO enrichment analysis of these 9857 genes showed that a handful of genes were associated with cellular response to heat, DNA damage and repair, cell cycle and cell death in cancer cells treated with hyperthermia (Fig. 4D and Table S5). Figure 4C indicated that the expression of a great number of the member of heat shock protein family, such as HSP90AA1, HSP90AA2P, HSP90AA4P, HSP90AB1, HSP90B1, HSPA13, HSPA4, HSPA4L, HSPA8, HSPA9P1, HSPB11, HSPD1, HSPD1P1, HSPE1, HSPE1-MOB4 and HSPE1P2, was significantly up-regulated in hyperthermia-treated NPC cells, while the decreased expression of Cirbp was observed in heat-treated cells. Collectively, our data demonstrate that hyperthermia treatment appreciably inhibits Cirbp expression in NPC cells, which led us to reasonably speculate that Cirbp silencing might induce the sensitization of NPC cells and cancer stem-like cells to hyperthermia.

### Cirbp suppression by RNAi significantly improved the sensitivity of NPC cells and cancer stem‑like cells to hyperthermia in vitro

To test above-mentioned hypothesis, we first evaluated the effects of Cirbp silencing by RNAi (Fig. S6) on the sensitivity of NPC cells and cancer stem-like cells to hyperthermia in vitro by CCK-8 assay (Fig. 4E), colony formation assay (Fig. 4F and Fig. S7A) and tumorsphere formation assay (Fig. 4G and Fig. S7B). The Cirbp-shRNA specifically knocked down endogenous Cirbp mRNA (Fig. S6A) and protein (Fig. S6B) expression in CNE2, SUNE1 and HONE1-EBV cells. Our results revealed that Cirbp knockdown led to significant cell viability inhibition (Fig. 4E), colony formation suppression (Fig. 4F and Fig. S7A) and tumorsphere formation inhibition (Fig. 4G and Fig. S7B), similar to those induced by hyperthermia treatment, suggesting that down-regulating Cirbp in NPC cells might mimic the stress response the cells experience when exposed to heat treatment. More importantly, treatment with combined hyperthermia and shRNA-mediated Cirbp silencing resulted in a substantial reduction in cell survival ability (Fig. 4E), colony formation suppression (Fig. 4F and Fig. S7A) and tumorsphere formation inhibition (Fig. 4G and Fig. S7B), as compared to thermotherapy alone. Together, these findings clearly demonstrate that Cirbp inhibition by RNAi significantly improve the thermosensitivity of NPC cells and cancer stem-like cells in vitro.

### Cirbp overexpression counteracted the tumor‑killing effects of hyperthermia on NPC cells and cancer stem‑like cells in vitro

**Fig. 5:**
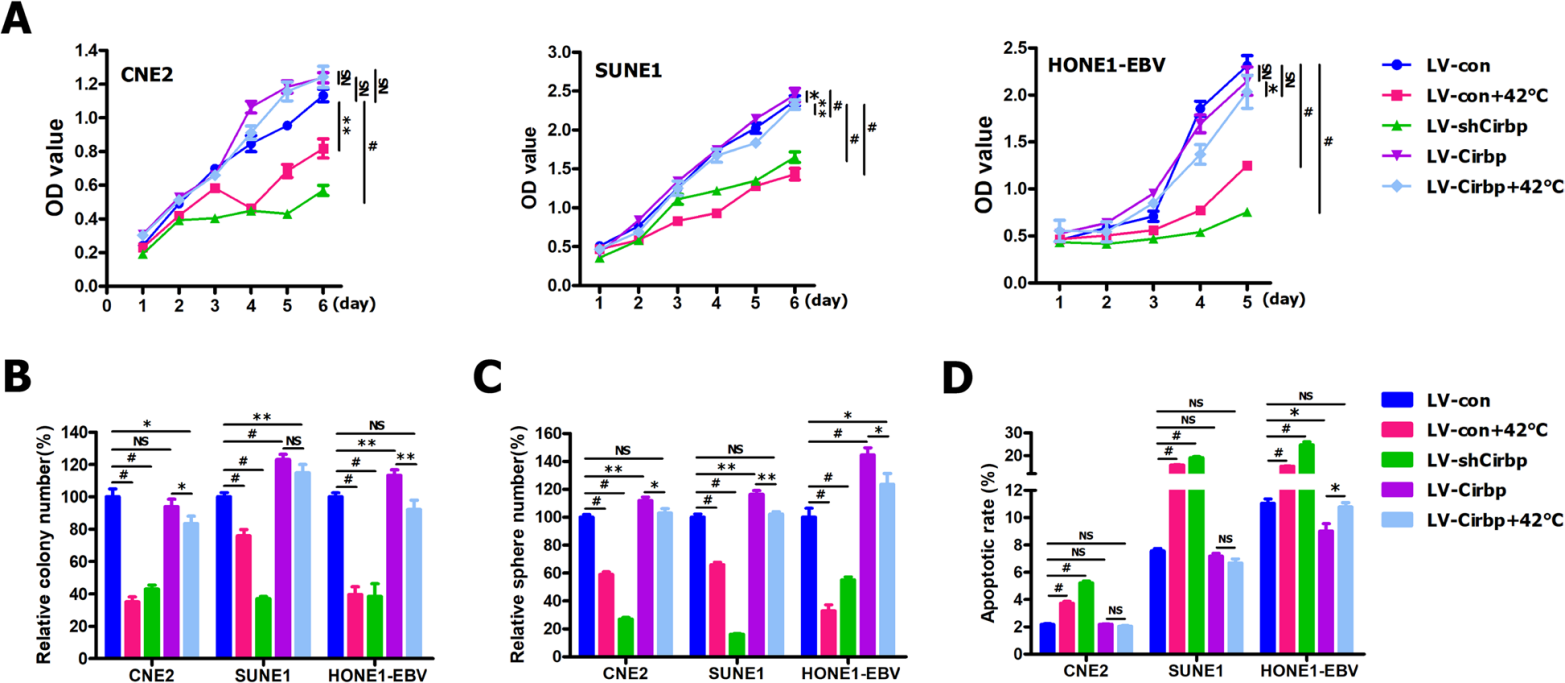
Exogenous expression of Cirbp counteracted the tumor-killing effect of hyperthermia on NPC cells and cancer stem-like cells in vitro. **A-D** CCK-8 assay **A**, colony formation assay **B**, tumor sphere formation assay **C** and AnnexinV/PI apoptosis assay **D** were performed in Cirbp-expressing NPC cells treated with or without hyperthermia at 42 °C for 30 min

Next, we further elucidated the effects of Cirbp overexpression on the sensitization of NPC cells and cancer stem-like cells to hyperthermia in vitro by CCK-8 assay (Fig. 5A), colony formation assay (Fig. 5B) and tumorsphere formation assay (Fig. 5C). The Cirbp transgene was successfully over-expressed in CNE2, SUNE1 and HONE1-EBV cells (Fig. S6). As shown in Fig. 5A, CCK-8 assay revealed that the ectopic expression of Cirbp in CNE2, SUNE1 and HONE1-EBV cells had no significant effect on cell viability, as compared with control cells (i.e., LV-con). Moreover, our results from colony formation assay (Fig. 5B and Fig. S8A) showed that exogenous expression of Cirbp had no statistically significant effect on CNE2 cell growth, and had a slight, but statistically significantly growth-promoting impacts on cell proliferation of SUNE1 and HONE1-EBV cells in vitro, as compared with control cells (i.e., LV-con). More importantly, CCK-8 assay (Fig. 5A) indicated that there was no significant difference in cell survival of CNE2, SUNE1 and HONE1-EBV cells between LV-Cirbp + 42 °C cells and LV-Cirbp cells. Additionally, colony formation assay showed that there was no statistically significant difference in cell proliferation of SUNE1 cells between LV-Cirbp + 42 °C cells and LV-Cirbp cells (Fig. 5B and Fig. S8A). As shown in Fig. 5B and Fig. S8A, heating treatment reduced colony number in Cirbp-expressing CNE2 and HONE1-EBV cells by about 10.50% (for CNE2 cells) and 21.10% (for HONE1-EBV cells) compared to LV-Cirbp cells, whereas LV-con + 42 °C cells displayed a substantial decrease in colony number by about 65.00% (for CNE2 cells) and 60.39% (for HONE1-EBV cells), as compared to LV-con cells. Collectively, these aforementioned findings clearly suggest that ectopic expression of Cirbp completely or mostly rescued hyperthermia-induced decrease in cell viability and cell growth in vitro.

In addition, our data from tumorsphere formation assay (Fig. 5C and Fig. S8B, C, D) demonstrated that the enforced expression of Cirbp had a slight, but statistically significantly growth-promoting effect on tumorsphere formation of CNE2 and SUNE1 cells, and displayed a significantly positive impacts on tumorsphere formation of HONE1-EBV cells, as compared with control cells (i.e., LV-con). As shown in Fig. 5C and Fig. S8B, C, D, heating treatment significantly reduced tumorsphere number in Cirbp-expressing CNE2, SUNE1 and HONE1-EBV cells by about 8.93% (for CNE2 cells), 14.21% (for SUNE1 cells) and 21.07% (for HONE1-EBV cells) compared to LV-Cirbp cells, whereas LV-con + 42 °C cells displayed a substantial decrease in tumorsphere number by about 41.01% (for CNE2 cells), 34.09% (for SUNE1 cells) and 67.07% (for HONE1-EBV cells), as compared to LV-con cells. Therefore, these aforementioned results clearly suggest that re-expression of Cirbp completely or mostly rescues hyperthermia-induced reduction in tumorsphere formation efficiency, indicating that re-expression of Cirbp induces hyperthermia resistance.

### Cirbp knockdown greatly promoted cell apoptosis and thus substantially enhanced the sensitivity of NPC cells to hyperthermia

To address whether Cirbp silencing-induced sensitization of NPC cells and cancer stem-like cells to hyperthermia in vitro is due to apoptosis, flow cytometry for the apoptosis assay was performed. In this study, we observed that hyperthermia treatment alone had a slight or significant impact on apoptosis induction, compared to sham-treated cells (i.e., LV-shSCR) (Fig. 4H and Fig. S7C) or (i.e., LV-con) (Fig. 5D and Fig. S8E). Moreover, Cirbp overexpression had minimal effect on apoptosis compared to sham-treated cells (i.e., LV-con) (Fig. 5D and Fig. S8E). However, Cirbp inhibition by RNAi had pronounced apoptosis-promoting effect on CNE2, SUNE1 and HONE1-EBV cells (Figs. 4H and 5D, Fig. S7C and Fig. S8E). Furthermore, the proportion of apoptotic cells of Cirbp knockdown plus 42 °C group increased significantly compared to those of the control group (LV-shSCR), shSCR + 42 °C group or LV-shCirbp group (Fig. 4H and Fig. S7C). Collectively, our results show that RNAi-mediated Cirbp suppression can greatly promote cell apoptosis, and thus substantially increase the sensitivity of cancer cells to hyperthermia.

To further identify genes involved in cell apoptosis and cell survival, we performed RNA-seq in shSCR- and shCirbp-expressing SUNE1 cells. Comparing shCirbp-expressing cells to control cells, differentially expressed genes were identified (Fig. 4I and Table S6) and classified using GO categories (Fig. 4J and Table S7) and KEGG pathway (Fig. 4J and Table S8). All GO terms representing biological processes listed in Fig. 4J and Table S7 were related to cell apoptosis, cell death and cell survival. Moreover, the functional classification of the differentially expressed mRNA transcripts based on KEGG pathway analysis also demonstrated that the downregulated genes in shCirbp-expressing SUNE1 cells are highly associated with PI3K-Akt signaling pathway and cGMP-PKG signaling pathway (Fig. 4J and Table S8). Together, these results from GO annotation and pathway enrichment analysis of differentially expressed genes illustrate a significant enrichment for selected genes with functions typically associated with cell apoptosis, cell death and cell survival, indicating that these altered genes involved in cell survival could be responsible, or contribute to the substantially increased sensitivity of shCirbp-expressing cancer cells to hyperthermia.

### ThermomiR‑377‑3p improved the sensitivity of NPC cells and cancer stem‑like cells to hyperthermia in vitro by directly suppressing Cirbp expression

**Fig. 6:**
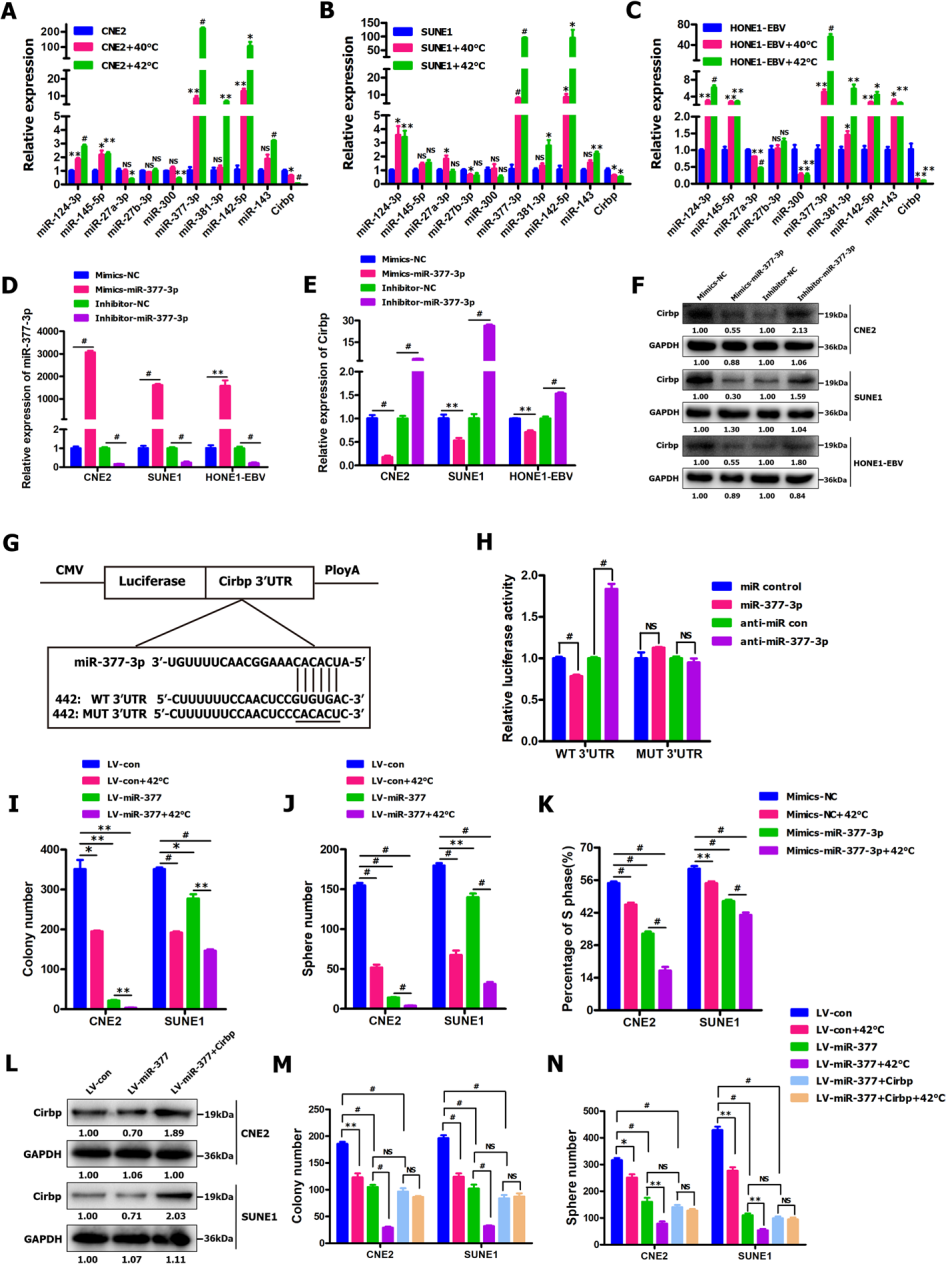
ThermomiR-377-3p improved the sensitivity of NPC cells and cancer stem-like cells to hyperthermia in vitro by directly suppressing Cirbp expression. **A-C** qRT-PCR assay for detecting the expression of selected miRNAs of which Cirbp might be a potential target gene in the indicated NPC cells treated with or without hyperthermia at 40 °C and 42 °C for 30 min. ThermomiRs (i.e., miR-143 and miR-142-5p) were used as positive controls. **D**, **E** qRT-PCR assay for detecting the expression of miR-377-3p **D** and Cirbp **E** in NPC cells transiently transfected with miR-377-3p mimics or inhibitor. **F** Western blot was employed to detect Cirbp expression in NPC cells transiently transfected with miR-377-3p mimics or inhibitor. **G** Diagram of 3’-UTR-WT and 3’-UTR-MUT of Cirbp containing reporter constructs. **H** Luciferase reporter assays in HEK293T cells co-transfected with WT or MUT 3’-UTR and miRNAs as indicated. **I**, **J** Colony formation assay **I** and tumor sphere formation assay **J** were performed in miR-377-expressing NPC cells treated with or without hyperthermia at 42 °C for 30 min. **K** EdU assay was performed in NPC cells transiently transfected with miR-377-3p mimics and then treated with or without hyperthermia at 42 °C for 30 min. **L** Western blot was employed to detect Cirbp expression in miR-377- and Cirbp-expressing NPC cells. **M**, **N** Colony formation assay **M** and tumor sphere formation assay **N** were performed in miR-377- and Cirbp-expressing NPC cells treated with or without hyperthermia at 42 °C for 30 min

miRNAs are being considered as potential therapeutic targets for various diseases, including hepatitis and cancers [9, 20, 28, 80, 91, 133]. In addition, the previous study indicated that some miRNAs (i.e., miR-142-5p and miR-143) were identified to belong to temperature-sensitive miRNAs (termed thermomiRs) [123]. Against this background, we intend to find out thermomiRs of which Cirbp might be a potential target gene. Based on bioinformatics prediction softwares (i.e., TargetScan, miRDB, PicTar and RNA22), we predicted seven miRNAs (i.e. miR-124-3p, miR-145-5p, miR-27a-3p, miR-27b-3p, miR-300, miR-377-3p and miR-381-3p) of which Cirbp might be a potential target gene. Subsequently, qRT-PCR assay was employed to detect the expression levels of the aforementioned miRNAs in hyperthermia-treated NPC cells. qRT-PCR analysis revealed the significantly elevated expression of thermomiRs (i.e., miR-143 and miR-142-5p used as positive controls) and two selected miRNAs (i.e., miR-377-3p and miR-381-3p), and the remarkably reduced expression of Cirbp in all of three NPC cell lines (CNE2, SUNE1 and HONE1-EBV) treated with hyperthermia at 40 °C or 42 °C (Fig. 6A, B, C), whereas qRT-PCR assay didn’t demonstrate the regular expression changes of other five miRNAs (i.e. miR-124-3p, miR-145-5p, miR-27a-3p, miR-27b-3p and miR-300) in three NPC cell lines treated with hyperthermia at both 40 °C and 42 °C (Fig. 6A, B, C), which prompted us to focus on two thermomiRs (i.e., miR-377-3p and miR-381-3p) for further study.

Our further study derived from qRT-PCR and Western blot illustrated that miR-377-3p (Fig. 6D, E, F), but not miR-381-3p (Fig. S9), negatively regulated Cirbp expression in all of three NPC cell lines (CNE2, SUNE1 and HONE1-EBV). Next, we further carried out luciferase reporter assay to determine whether miR-377-3p can directly target the 3’-UTR of Cirbp. The target sequence of Cirbp 3’-UTR (3’-UTR-WT) or the mutant sequence (3’-UTR-MUT) was cloned into a luciferase reporter vector (Fig. 6G). HEK293T cells were then transfected with WT or MUT 3′-UTR vector and miR-377-3p mimics. The results showed a significant decrease of luciferase activity when compared with miR control (Fig. 6H; *P* < 0.001). The activity of luciferase was unaffected by a simultaneous cotransfection with miR-377-3p and MUT 3’-UTR vector (Fig. 6H). Furthermore, cotransfection with anti-miR-377-3p and WT 3′-UTR vector in HEK293T led to a significant increase of luciferase activity (Fig. 6H; *P* < 0.001). Together, all these results strongly suggest that Cirbp is a direct target of miR-377-3p.

Next, we further elucidated the effects of miR-377-3p overexpression on the sensitization of NPC cells and cancer stem-like cells to hyperthermia in vitro by colony formation assay (Fig. 6I), tumorsphere formation assay (Fig. 6J) and EdU assay (Fig. 6K). The miR-377 transgene was successfully over-expressed in CNE2 and SUNE1 cells (Fig. S10A, B). Our results revealed that miR-377-3p overexpression led to significant colony formation suppression (Fig. 6I and Fig. S10C), tumorsphere formation inhibition (Fig. 6J and Fig. S10D) and cell growth inhibition (shown by EdU assay) (Fig. 6K and Fig. S10E), similar to those induced by hyperthermia treatment, suggesting that ectopic expression of miR-377-3p in NPC cells might mimic the stress response the cells experience when exposed to heat treatment. More importantly, treatment with combined hyperthermia and miR-377-3p overexpression resulted in a substantial reduction in colony formation suppression (Fig. 6I and Fig. S10C), tumorsphere formation inhibition (Fig. 6J and Fig. S10D) and cell growth inhibition (shown by EdU assay) (Fig. 6K and Fig. S10E), as compared to thermotherapy alone. Together, these findings clearly demonstrate that the enforced expression of miR-377-3p significantly improve the thermosensitivity of NPC cells and cancer stem-like cells in vitro, similar to those induced by RNAi-mediated Cirbp inhibition plus hyperthermia treatment.

To elucidate whether the thermosensitivity-improved effects of miR-377-3p overexpression was mediated by repression of Cirbp in NPC cells, we further evaluated whether ectopic expression of Cirbp could rescue the thermosensitivity-improved effects of miR-377-3p. To this end, exogenous expression of Cirbp was attained in miR-377-expressing NPC cells (Fig. 6L). We found that ectopic expression of Cirbp significantly rescued miR-377-3p-induced colony formation suppression (Fig. 6M and Fig. S10F) and tumorsphere formation inhibition (Fig. 6N and Fig. S10G) under hyperthermia condition. Collectively, thermomiR-377-3p improves the sensitivity of NPC cells and cancer stem-like cells to hyperthermia in vitro by directly suppressing Cirbp expression.

### Sensitization of tumor xenografts to hyperthermia by Cirbp silencing in vivo

**Fig. 7:**
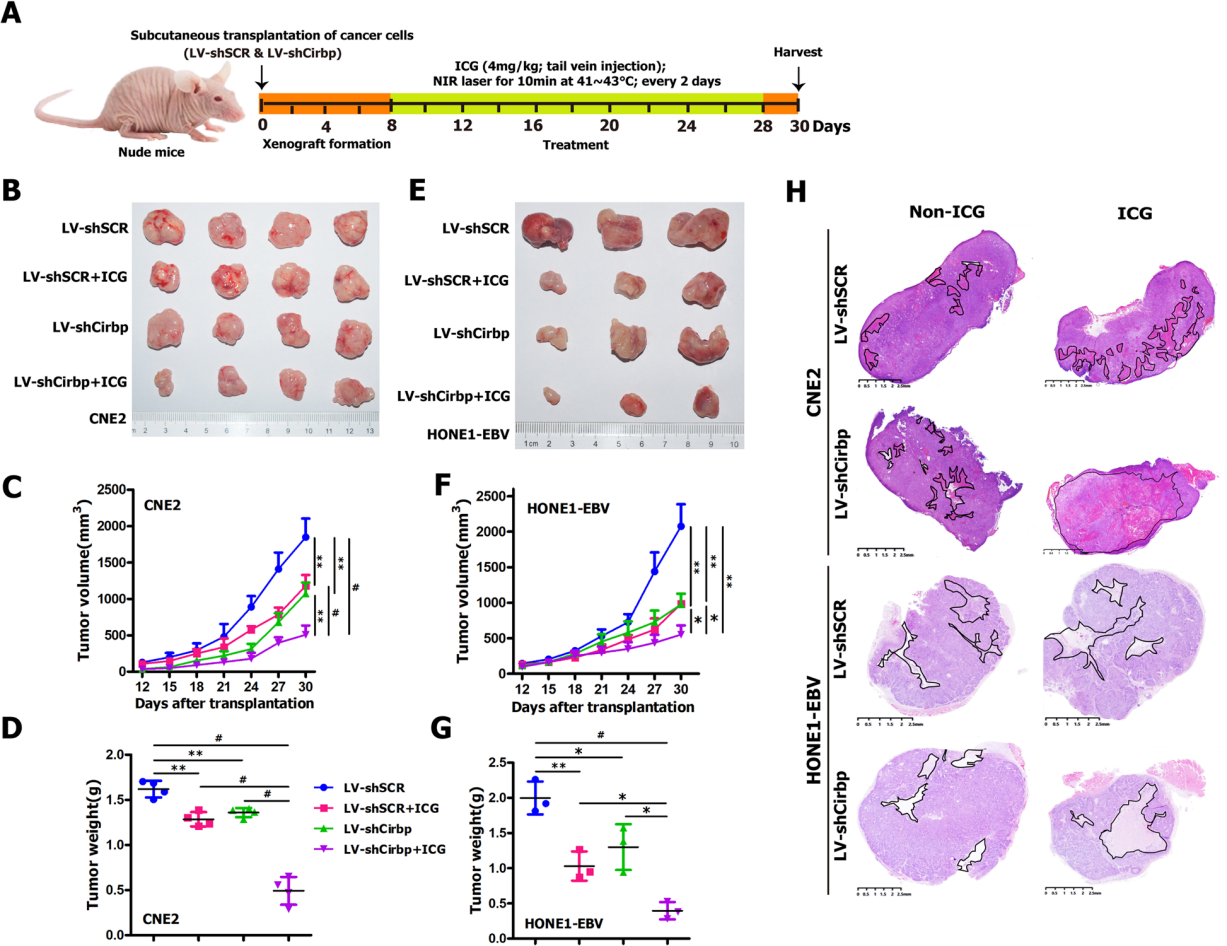
Cirbp silencing-induced sensitization of subcutaneous tumor xenografts to hyperthermia by local thermal ablation with ICG and an NIR laser in vivo. **A** Schematic representation of the experimental design of hyperthermia treatment in nude mice harboring subcutaneous tumor xenografts formed by CNE2 or HONE1-EBV cells. **B**, **E** Representative images of stripped xenograft tumors formed by CNE2 **B** and HONE1-EBV **E** cells at the end of hyperthermia therapy (*n* = 3-4 mice/group). **C**, **F** The tumor growth curve (*n* = 3-4 mice/group). **D**, **G** Tumor weight (*n* = 3-4 mice/group). **H** Representative pictures of H&E staining of stripped xenograft tumors (showed in Fig. 7B, E)

Next, we further evaluated the in vivo effects of Cirbp suppression on the sensitization of NPC cells to hyperthermia and on tumor growth in subcutaneous xenograft tumor mouse model of NPC cells. Firstly, to this end, BALB/C nude mice were selected and the xenograft tumor models were established with NPC cell lines (i.e., CNE2, HONE1-EBV and SUNE1 cells) by subcutaneous injection, according to standard procedures described in the section of Materials and Methods. Figure 7A presents the experimental schedule for in vivo animal study. Indocyanine green (ICG) is a photothermal agent, photosensitizer, and fluorescence imaging probe which shows specific accumulation in cancer cells [5, 95, 98, 115, 131, 135]. In this study, we developed a photodynamic therapy (PDT) (i.e., ICG-NIR therapy) using ICG and near-infrared (NIR) laser as an anti-tumor therapy for NPC. As showed in Fig. 7A, ICG-NIR-mediated PDT was employed to further identify the in vivo effects of Cirbp inhibition on the therapeutic efficacy of thermotherapy. As indicated in Fig. 7A, 30 min before NIR laser treatment, we intravenously injected ICG into tumor-bearing nude mice, and subsequently we treated tumors with local hyperthermia (41 °C–43 °C) using an NIR laser at 808 nm for 10 min. Repeated ICG-NIR irradiation with a 808 nm was carried out for 10 min every 2 days. As expected, compared to control group (i.e., LV-shSCR), in vivo Cirbp suppression by RNAi in CNE2 and HONE1-EBV cell-derived xenografts resulted in a dramatic reduction in tumor size (Fig. 7B, E), tumor volume (Fig. 7C, F) and tumor weight (Fig. 7D, G), similar to those induced by hyperthermia treatment alone (i.e., LV-shSCR + ICG) (Fig. 7B-G), indicating that down-regulating Cirbp in subcutaneous tumor xenograft formed by NPC cells mimics the stress response the cells experience when exposed to local hyperthermia treatment. More importantly, the combination treatment with Cirbp silencing and local hyperthermia (i.e., LV-shCirbp + ICG) led to the dramatic inhibition of tumor growth, as shown by substantially reduced tumor size (Fig. 7B, E), tumor volume (Fig. 7C, F) and tumor weight (Fig. 7D, G), as compared with Cirbp knockdown or hyperthermia alone. Therapeutic benefit from Cirbp inhibition plus hyperthermia combination treatment was also attained in subcutaneous xenograft model of SUNE1 cells (Fig. S11A). Furthermore, H&E staining indicated that there were large necrotic areas within CNE2, HONE1-EBV and SUNE1 cell-derived tumor xenograft tissues in local hyperthermia alone group, similar to those induced by in vivo Cirbp suppression by RNAi (Fig. 7H and Fig. S11B). More importantly, the combination treatment of Cirbp silencing and local hyperthermia leads to considerably necrotic area within xenograft tumor tissues, as compared with Cirbp inhibition or thermotherapy alone (Fig. 7H and Fig. S11B). Taken together, these results demonstrate that Cirbp silencing in vivo sensitizes NPC xenograft tumor to local hyperthermia treatment, and thus substantially boosts anti-tumor killing effects of hyperthermia against NPC cells and cancer stem-like cells in vivo.

### Exogenous expression of Cirbp counteracted the tumor‑killing effect of hyperthermia against NPC cells and cancer stem‑like cells in vivo

**Fig. 8:**
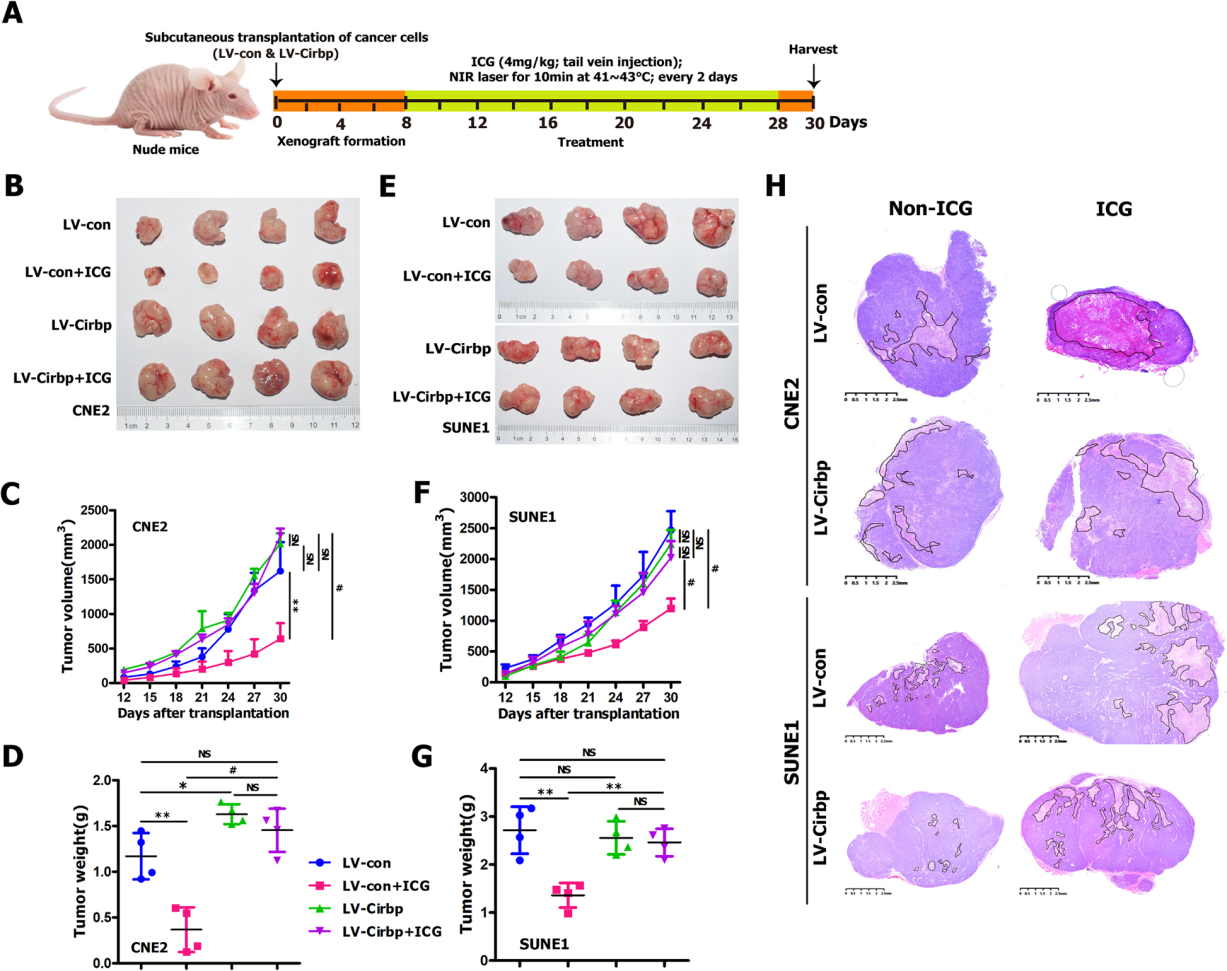
Ectopic expression of Cirbp counteracted the tumor-killing effect of hyperthermia on NPC cells and cancer stem-like cells in vivo. **A** Schematic representation of the experimental design of hyperthermia treatment in nude mice bearing subcutaneous tumor xenografts formed by CNE2 or SUNE1 cells. **B**, **E** Representative images of stripped xenograft tumors formed by CNE2 **B** and SUNE1 **E** cells at the end of hyperthermia therapy (*n* = 4 mice/group). **C**, **F** The tumor growth curve (*n* = 4 mice/group). **D**, **G** Tumor weight (*n* = 4 mice/group). **H** Representative pictures of H&E staining of stripped xenograft tumors (showed in Fig. 8B,E)

Subsequently, we further identified the in vivo effects of re-expression of Cirbp on the sensitization of NPC cells (i.e., CNE2 and SUNE1 cells) to hyperthermia and on tumor growth of NPC cell-derived xenografts (Fig. 8). As expected, in vivo local hyperthermia alone (i.e., LV-con + ICG) significantly decreased tumor size (Fig. 8B, E), tumor volume (Fig. 8C, F) and tumor weight (Fig. 8D, G) compared to mock-treated group (i.e., LV-con). Moreover, ectopic expression of Cirbp in CNE2 (Fig. 8B, C, D) and SUNE1 (Fig. 8E,F,G) cells had little effect on tumor xenograft growth, as compared with control cells (i.e., LV-con). More interestingly, we clearly observed there was no statistically significant difference in tumor size (Fig. 8B,E), tumor volume (Fig. 8C, F) and tumor weight (Fig. 8D, G) of tumor xenografts formed by CNE2 or SUNE1 cells between LV-Cirbp + ICG group and LV-Cirbp group, suggesting that exogenous expression of Cirbp completely rescued hyperthermia-induced significant inhibition in tumor xenograft growth. Additionally, as expected, histological examinations after local hyperthermia treatment alone (i.e., LV-con + ICG group) revealed large necrotic areas, as compared with control group (i.e., LV-con) (Fig. 8H). More interestingly, we clearly found there was no significant difference in necrotic area tumor xenograft formed by CNE2 or SUNE1 cells between LV-Cirbp + ICG group and LV-Cirbp group, suggesting that exogenous expression of Cirbp completely or mostly compromised hyperthermia-induced necrosis (Fig. 8H). Taken together, our these findings evidently illustrate that ectopic expression of Cirbp completely or mostly counteracts the sensitivity of cells to hyperthermia, and thus completely or mostly neutralized the anti-tumor activity of hyperthermia against NPC cells and cancer stem-like cells in vivo, suggesting that Cirbp overexpression causes hyperthermia resistance.

### Cirbp positively modulated the resistance of CSC‑like cells to hyperthermia

**Fig. 9:**
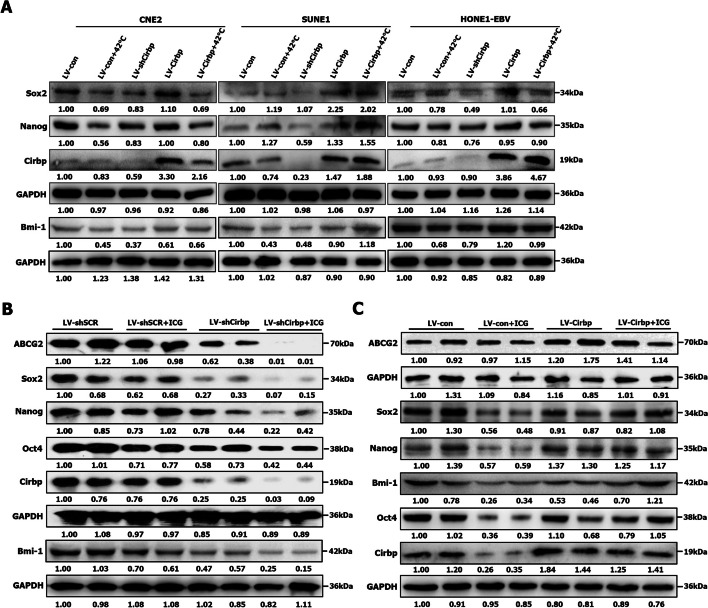
Cirbp positively regulated the resistance of CSC-like cells to hyperthermia. **A** Western blot analysis of stemness-related gene expression in Cirbp-expressing and shCirbp-expressing NPC cells treated with or without hyperthermia at 42 °C for 30 min. **B** Western blot analysis of stemness-related gene expression in xenograft tumors (showed in Fig. 7B) formed by CNE2 cells. **C** Western blot analysis of stemness-related gene expression in xenograft tumors (showed in Fig. 8B) formed by CNE2 cells

CSCs are thought to be responsible for the therapeutic resistance to conventional treatments (including radiotherapy, chemotherapy, immunotherapy and thermal therapy) [3, 7, 8, 25, 29, 43, 52, 76, 77, 81]. Considering the importance of CSCs in the maintenance of therapeutic resistance, we further evaluated the effects of Cirbp on the sensitivity or the resistance of cancer stem-like cells to hyperthermia by tumorsphere formation assay (Figs. 4G and 5C) and detecting stem cell-related gene expression (Fig. 9A, B, C). Our results revealed that Cirbp knockdown alone led to significant tumorsphere formation inhibition (Fig. 4G and Fig. S7B), similar to those induced by hyperthermia treatment alone, indicating that down-regulating Cirbp significantly reduces the self-renewal ability of cancer stem-like cells. More importantly, treatment with combined hyperthermia and siRNA-mediated Cirbp silencing resulted in a substantial reduction in tumorsphere formation (Fig. 4G and Fig. S7B), as compared to thermotherapy or LV-shCirbp group alone.

Subsequently, we further evaluated the stemness by detecting stem cell-related gene expression. Western blot analysis revealed that heat treatment (i.e., LV-con + 42 °C group, LV-con + ICG group or LV-shSCR + ICG group) or Cirbp inhibition by RNAi alone resulted in the remarkable downregulation of stem cell-related markers (i.e., Sox2, Nanog, Oct4, ABCG2 or Bmi-1) in NPC cells (i.e., CNE2, SUNE1 and HONE1-EBV cells) (Fig. 9A) and in CNE2 cell-derived xenografts (Fig. 9B and C), as compared with those in control cells (i.e., LV-con group) or in control xenografts [i.e., LV-shSCR group (Fig. 9B) or LV-con group (Fig. 9C)], suggesting that hyperthermia or Cirbp depletion efficiently suppresses the stemness of NPC cells in vitro and in vivo. Moreover, treatment with combined hyperthermia and Cirbp silencing by RNAi dramatically reduced expression or led to almost undetectable expression levels of the indicated stem cell-related markers in whole xenograft tumor lysates from CNE2 cells (Fig. 9B), as compared to those of hyperthermia or Cirbp inhibition alone. Taken together, these results indicate that Cirbp suppression-hyperthermia combination treatment efficiently attenuates the stemness of NPC cells, which thereby contributes to significantly improving the sensitivity of cancer stem-like cells to hyperthermia.

As mentioned above, ectopic expression of Cirbp completely or mostly rescues hyperthermia-induced reduction in tumorsphere formation efficiency (Fig. 5C and Fig. S8B, C, D). In addition, we observed that heat treatment alone (i.e., LV-con + ICG group) led to the remarkable downregulation of stem cell-related markers (i.e., Nanog, Sox2, Oct4, ABCG2 or Bmi-1) in CNE2 cell-derived xenografts (Fig. 9C), as compared with those in control xenografts [i.e., LV-con group (Fig. 9C)], suggesting that hyperthermia efficiently suppresses the stemness of NPC cells in vivo. The enforced expression of Cirbp alone had little impact on the expression of stem cell-related markers (i.e., Nanog and Bmi-1) or slightly upregulated the expression of Sox2, Oct4 and ABCG2 in whole xenograft tumor lysates from CNE2 cells (Fig. 9C), as compared to those of control group (i.e., LV-con group). Finally, hyperthermia led to a slight down-regulation of indicated stem cell-related genes in tumor xenografts formed by Cirbp-expressing CNE2 cells, as compared to those in LV-Cirbp group (Fig. 9C), whereas the expression levels of stem cell-related genes (i.e., Sox2 or ABCG2) in LV-Cirbp + ICG group were slightly higher than those in LV-con + ICG group. Thus, these data clearly suggest that ectopic expression of Cirbp partially counteracts hyperthermia-induced decrease in stem cell-related gene expression. All in all, re-expression of Cirbp completely or mostly compromises hyperthermia-induced reduction in the stemness of NPC cells, which thereby contributes to hyperthermia resistance.

### Cirbp silencing‑induced inhibition of DNA repair and increase in cell death contribute to hyperthermic sensitization

Subsequently, we intended to further investigate the underlying mechanisms by which Cirbp regulates thermosensitivity. As a stress-induced protein, Cirbp is initially described as a DNA damage-induced transcript (A18 hnRNP) [73], while Cirbp has been implicated in DNA damage and repair [10, 51, 57, 73]. Therefore, these above-mentioned findings led us to reasonably infer that Cirbp-mediated DNA damage and repair might be involved in the underlying mechanisms by which Cirbp regulates the sensitivity of cancer cells to hyperthermia.

**Fig. 10:**
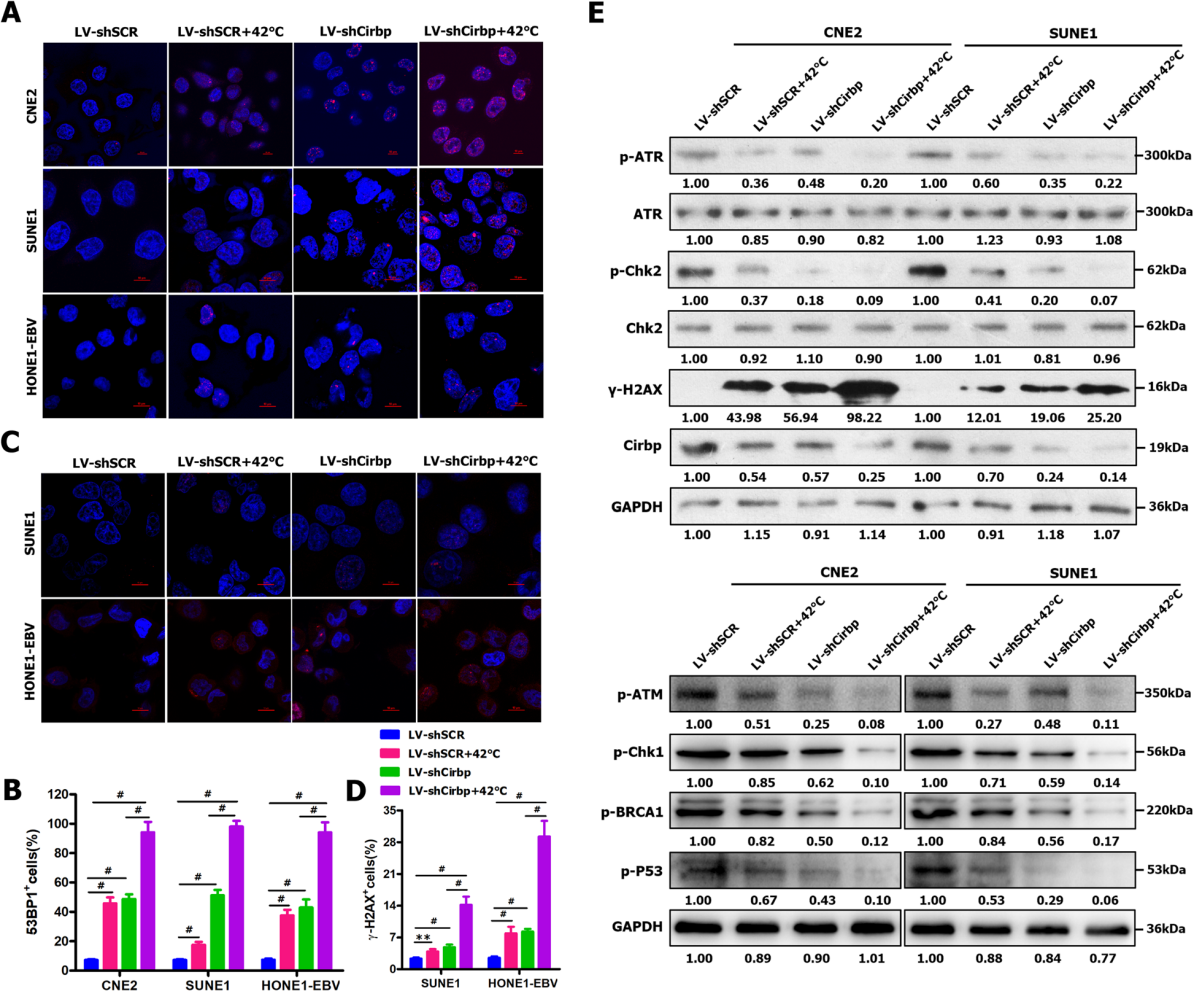
Cirbp silencing-induced inhibition of DNA damage repair. **A**, **B** Representative pictures of 53BP1 staining (red) **A** and quantification of the fraction of 53BP1^+^ cells **B** in shCirbp-expressing NPC cells treated with or without hyperthermia at 42 °C for 30 min. **C**, **D** Representative pictures of γ-H2AX staining(red) **C** and quantification of the fraction of γ-H2AX^+^ **D** in shCirbp-expressing NPC cells treated with or without hyperthermia at 42 °C for 30 min. **E** Western blot assay was used to detect Cirbp, p-ATM, p-ATR, p-Chk1, p-Chk2, p-BRCA1, p-p53 and γ-H2AX in indicated cells

**Fig. 11:**
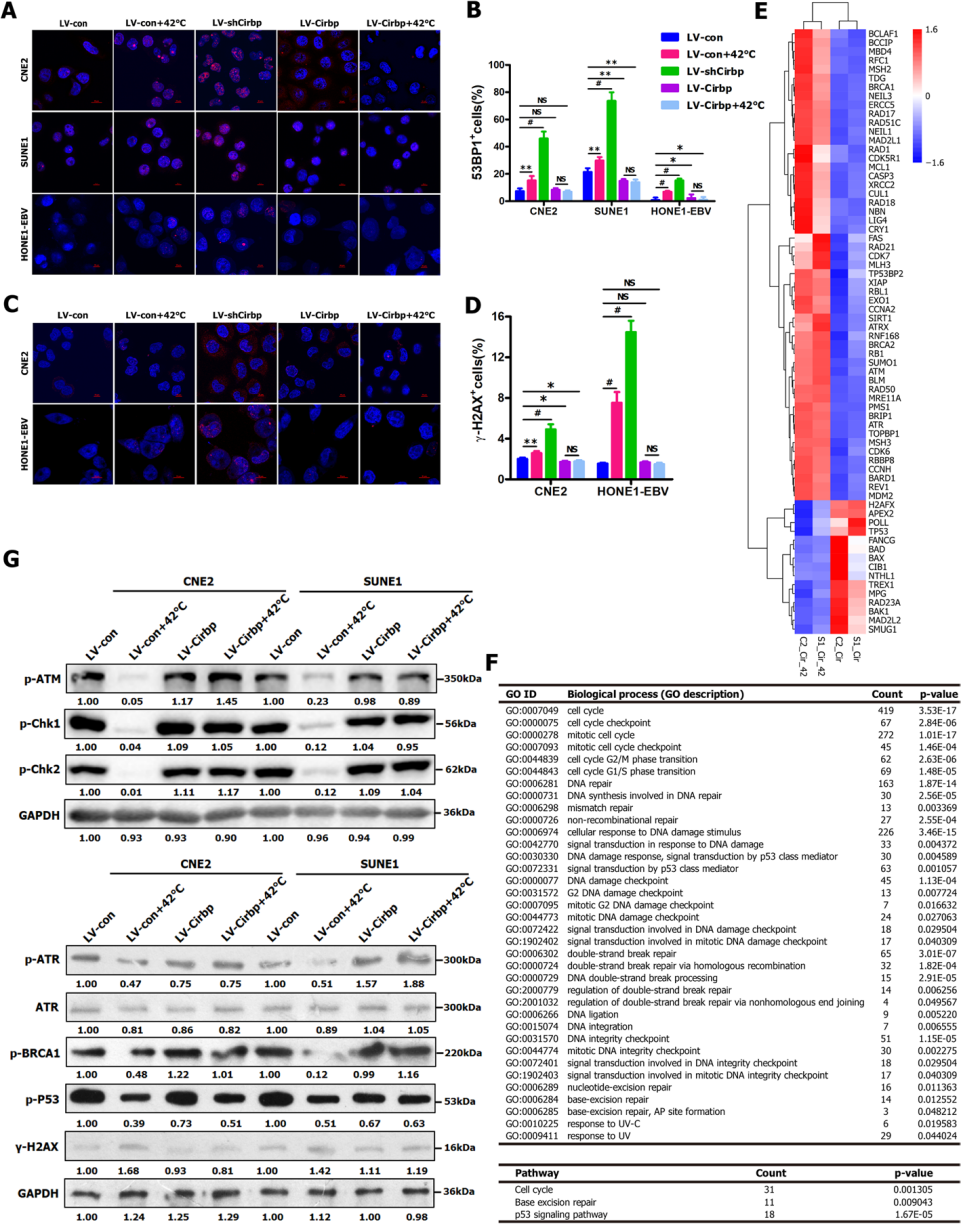
Ectopic expression of Cirbp counteracts the thermosensitivity of NPC cells by promoting DNA damage repair. **A**, **B** Representative pictures of 53BP1 staining(red) **A** and quantification of the fraction of 53BP1^+^ cells **B** in Cirbp-expressing NPC cells treated with or without hyperthermia at 42 °C for 30 min. **C**, **D** Representative pictures of γ-H2AX staining(red) **C** and quantification of the fraction of γ-H2AX^+^ **D** in Cirbp-expressing NPC cells treated with or without hyperthermia at 42 °C for 30 min. **E** Heatmap showing selected differentially expressed genes (see Table S10) involved in DNA damage and repair, and cell cycle in Cirbp-expressing CNE2 and SUNE1 cells. Right column lists the selected gene symbols. **F** GO and KEGG pathway analysis of up- and down-regulated genes (see Tables S11 and S12) involved in DNA damage and repair, and cell cycle in Cirbp-expressing CNE2 and SUNE1 cells. **G** Cirbp, p-ATM, p-ATR, p-Chk1, p-Chk2, p-BRCA1, p-p53 and γ-H2AX in indicated cells were determined by Western blot

Firstly, to further explore the impacts of Cirbp depletion on DNA damage and repair during thermotherapy, immunofluorescent staining was performed to examine the presence of DNA double-strand break (DSB) in indicated cells by assessing the formation of 53BP1(Fig. 10A, B) and γ-H2AX (Fig. 10C, D) foci. We observed that there were significantly more 53BP1-labeled CNE2, SUNE1 and HONE1-EBV cells in both LV-shSCR + 42 °C group and LV-shCirbp group, as compared to those of control group (i.e., LV-shSCR) (Fig. 10A, B). Moreover, 53BP1 exhibited nuclear foci in more cancer cells of LV-shCirbp + 42 °C group compared with those in LV-shSCR group, LV-shSCR + 42 °C group and LV-shCirbp group (Fig. 10A, B). As shown in Fig. 10C,D, compared to that in control cells (i.e., LV-shSCR), the percentage of γ-H2AX-positive cells (i.e., SUNE1 and HONE1-EBV cells) was much higher in shCirbp-expressing cells of LV-shCirbp group, similar to those induced by hyperthermia treatment alone. More importantly, our results displayed the higher percentage of γ-H2AX-positive cells in shCirbp-expressing SUNE1 and HONE1-EBV cells (especially HONE1-EBV cells) that underwent thermotherapy, as compared with those in LV-shSCR group, LV-shSCR + 42 °C group and LV-shCirbp group (Fig. 10C, D). Moreover, the protein levels of γ-H2AX were also quantified by Western blot. Compared to control cells [i.e., LV-shSCR group (Fig. 10E) or LV-con group (Fig. 11G)], NPC cells(i.e., CNE2 and SUNE1 cells) treated with hyperthermia [i.e., LV-shSCR + 42 °C group (Fig. 10E) or LV-con + 42 °C group (Fig. 11G)] had higher levels of γ-H2AX, while shCirbp-expressing cells also showed increased levels of γ-H2AX, as compared with control [i.e., LV-shSCR group (Fig. 10E)]. Importantly, the treatment with combined hyperthermia and shCirbp showed higher level of γ-H2AX, as compared with hyperthermia or Cirbp inhibition by RNAi alone (Fig. 10E). These above-mentioned findings from Western blot (Figs. 10E and 11G) are consistent with the results of the immunofluorescence (Fig. 10C, D). Thus, two indicators of DSBs suggest the presence of higher incidence of DNA damage in the combination treatment group. Furthermore, as described above, shCirbp-expressing cells (i.e., CNE2, SUNE1 and HONE1-EBV cells) treated with hyperthermia exhibited higher levels of apoptosis (Fig. 4H). Together, these data suggest that Cirbp silencing-induced inhibition of DNA damage repair and thus increase in cell death might contribute to hyperthermic sensitization.

Upon hyperthermia-induced DNA damage, tumor cells utilize two primarily distinct kinase signaling cascades to repair DSBs, including the ATM-Chk2 and ATR-Chk1 axes [30, 31, 40, 99, 107, 109, 135]. PARP-1-dependent recruitment of Cirbp promotes double-strand break repair and genome stability [10]. Recent study reported that Cirbp plays a crucial role in mediating the associations of MRN and ATM with chromatin [10]. Firstly, to gain more insight into the roles of hyperthermia and Cirbp knockdown alone or combined in the activation or inactivation of these DNA repair pathways, we detected the phosphorylation of ATM (p-ATM), Chk2 (p-Chk2), p53 (p-p53), ATR (p-ATR), Chk1 (p-Chk1) and BRCA1 (p-BRCA1) in shCirbp-expressing CNE2 and SUNE1 cells before and after heating treatment. Compared to control cells [i.e., LV-shSCR group (Fig. 10E)], Cirbp knockdown alone led to the significant reduction in the phosphorylation levels of ATM (p-ATM), Chk2 (p-Chk2), p53 (p-p53), ATR (p-ATR), Chk1 (p-Chk1) and BRCA1 (p-BRCA1) in CNE2 and SUNE1 cells (Fig. 10E), similar to those induced by hyperthermia treatment alone (Figs. 10E and 11G). As expected, treatment with combined hyperthermia and Cirbp silencing dramatically suppressed the phosphorylation levels of these above-mentioned proteins in indicated cells compared to hyperthermia or Cirbp inhibition alone (Fig. 10E). Overall, these findings indicate that Cirbp knockdown represses ATM-Chk2 and ATR-Chk1 pathways, and consequently decreases the DNA repair ability of NPC cells, ultimately enhancing thermosensitivity.

### Ectopic expression of Cirbp induced hyperthermia resistance through promoting DNA damage repair

Secondly, we further investigated the effects of the enforced Cirbp expression on DNA damage and repair during hyperthermia using immunofluorescent staining for nuclear foci of the protein 53BP1(Fig. 11A, B) and γ-H2AX (Fig. 11C, D). We observed that hyperthermia treatment alone led to the statistically significant formation of 53BP1 (Fig. 11B) and γ-H2AX (Fig. 11D) foci, whereas in the absence of stress, the ectopic expression of Cirbp in the indicated cells had little effect on the formation of 53BP1 (Fig. 11A, B) and γ-H2AX (Fig. 11C, D) foci, as compared with control group (i.e., LV-con alone). More interestingly, the formation of 53BP1 (Fig. 11B) and γ-H2AX (Fig. 11D) foci was observed in the two groups (i.e., LV-Cirbp group and LV-Cirbp + 42 °C group) at virtually equal and low levels, suggesting that ectopic expression of Cirbp counteracts hyperthermia-induced formation of 53BP1 and γ-H2AX foci in LV-Cirbp + 42 °C group. Furthermore, there was no significant difference in apoptotic cell rate between LV-Cirbp group and LV-Cirbp + 42 °C group (Fig. 5D). Collectively, these above-mentioned findings led us to reasonably infer that Cirbp overexpression might protect cancer cells against hyperthermia-induced DNA damage, which thereby contributes to hyperthermia resistance.

To fully understand the molecular basis that contribute to the Cirbp overexpression-induced hyperthermia resistance, we performed RNA-seq in Cirbp-expressing NPC cells and Cirbp-expressing NPC cells plus 42 °C. Comparing Cirbp-expressing NPC cells plus 42 °C to Cirbp-expressing NPC cells, a total of 4020 differentially expressed genes were identified (Fig. 11E, Fig. S12, Tables S9 and S10) and classified using GO categories (Fig. 11F and Table S11) and KEGG pathway (Fig. 11F and Table S12). In the present study, we found that the biological implications of up-regulated genes in Cirbp-expressing NPC cells plus 42 °C were significantly over-represented in GO biological processes related to DNA repair and cell cycle (Fig. 11E, F, Tables S9, S10 and S11), and KEGG pathway including p53 signaling pathway (Fig. 11, Tables S9, S10 and S12). Cirbp that is initially described as a DNA damage-induced transcript is a stress-induced protein [73], while PARP-1-dependent recruitment of Cirbp promotes double-strand break (DSB) repair and genome stability [10]. These above-mentioned findings from RNA-seq and published reports strongly support that ectopic expression of Cirbp activates DNA repair pathways in NPC cells under hyperthermia condition.

**Fig. 12:**
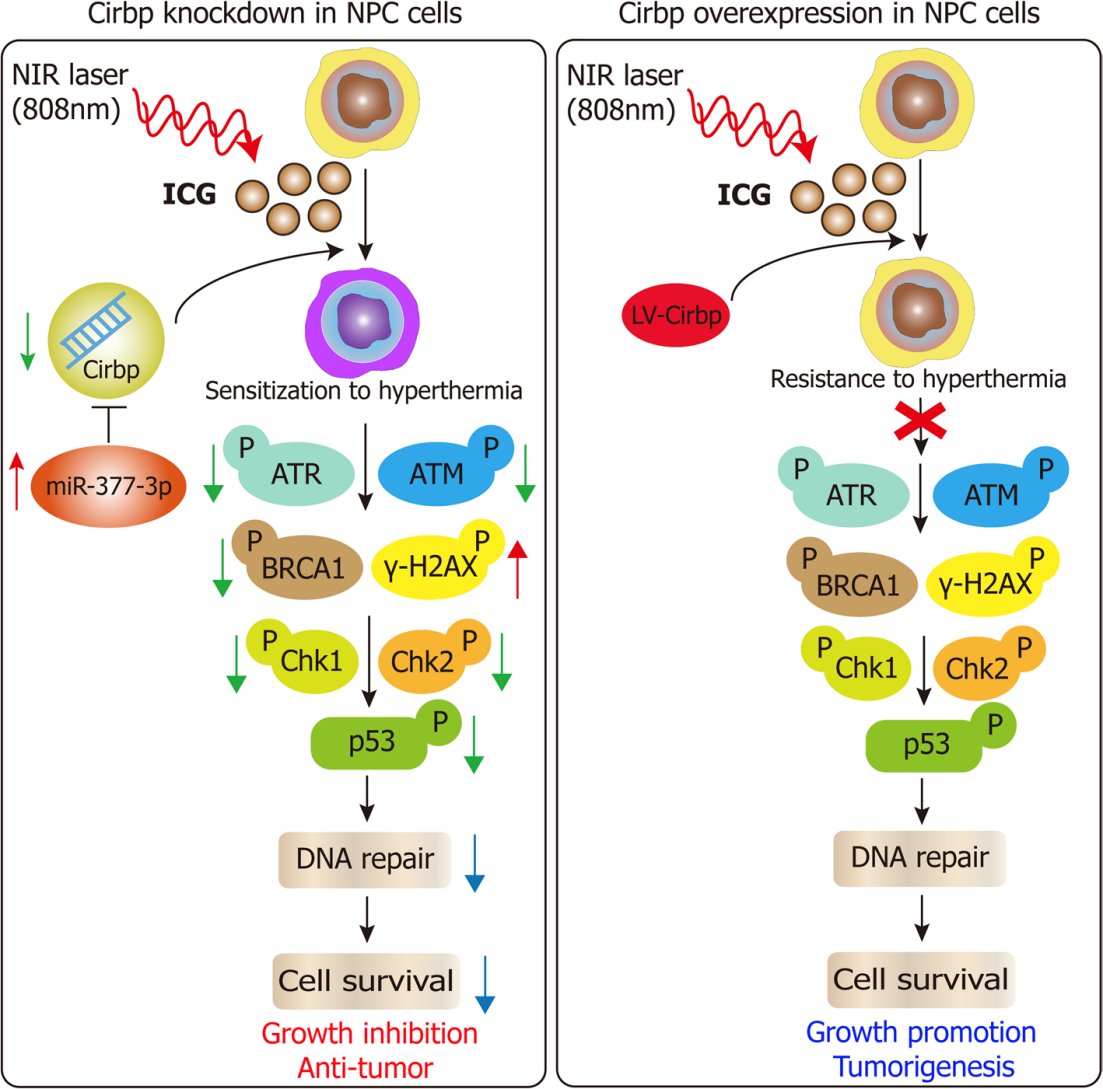
A proposed mechanism of Cirbp-mediated resistance and sensitization to hyperthermia

To gain additional insight into the effects of hyperthermia and Cirbp overexpression alone or combined on DNA damage repair pathways, we examined the changes in the aforementioned key proteins involved in the DNA repair pathways in Cirbp-expressing CNE2 and SUNE1 cells before and after heating treatment. We observed that hyperthermia treatment alone significantly reduced the phosphorylation levels of ATM (p-ATM), Chk2 (p-Chk2), p53 (p-p53), ATR (p-ATR), Chk1 (p-Chk1) and BRCA1 (p-BRCA1) in indicated cells (Fig. 11G). In the absence of stress, the ectopic expression of Cirbp in CNE2 and SUNE1 cells had little effect on the phosphorylation levels of these above-mentioned proteins involved in DNA repair pathways, as compared to those in control cells (i.e., LV-con group) (Fig. 11G). Moreover, compared to those in LV-Cirbp group, the phosphorylation levels of ATM, Chk2, p53, ATR and Chk1 were not significantly altered or slightly changed in LV-Cirbp + 42 °C group, whereas cells from LV-Cirbp + 42 °C group exhibited relatively higher activation of these two pathways compared to those in LV-con + 42 °C group (Fig. 11G). These aforementioned results clearly suggests that ectopic expression of Cirbp mostly or completely rescues hyperthermia-induced reduction in the phosphorylation levels of ATM, Chk2, p53, ATR and Chk1, which consequently reverses hyperthermia-induced reduction in DNA damage repair ability of cancer cells and increase in cell apoptosis, ultimately leading to increased thermoresistance and tumor growth (Fig. 12). Summarily, these data clearly suggest that Cirbp overexpression-induced promotion of DNA damage repair and decrease in cell death contributes to hyperthermia resistance.

Moreover, we further found that hyperthermia notably suppressed Cirbp protein expression in indicated heating-treated CNE2 and SUNE1 cells (Fig. 10E), which further validates these mentioned-above in vitro observations on the dramatically downregulated mRNA and protein expression of Cirbp in heating-treated NPC cells (Fig. 4A, B, C).

## Discussion

As a promising adjunctive therapy, hyperthermia will play an important role in multidiscipline therapy for cancer [2, 23, 45, 50]. However, the underlying molecular mechanisms involved in the response of tumor cells to thermal therapy still remain largely unknown. In this study, our findings uncover, for what we believe is the first time, that exogenous expression of Cirbp enhances hyperthermia resistance by promoting DNA damage repair in cancer cells, while Cirbp suppression is required for effective elimination of cancer cells and cancer stem-like cells by hyperthermia. This role of Cirbp is distinct from the established functions of Cirbp in RNA metabolism, circadian gene regulation and inflammatory response [4, 10, 35, 59, 61, 73, 137, 138].

Even though hyperthermia provides a promising therapeutic approach to anti-tumor therapy, huge efforts will still be required to fully examine the molecular mechanisms involved in tumor response to hyperthermia, which is critical to tremendously improve the clinical efficacy of hyperthermia by manipulating key pathways [67]. Several lines of evidence showed that the suppression of some genes or proteins, such as telomerase catalytic subunit TERT [1], AKT signaling [75], CTGF [33], HSP70 [19, 93], HSP90 [114] and HSP27 [79] significantly boosted the effects of hyperthermia-based anti-cancer treatments. Furthermore, introduction of constitutively active AKT in glioma stem cells (GSCs) compromised hyperthermic radiosensitization [75]. Collectively, the underlying mechanisms involved in tumor response to hyperthermia, especially involved in hyperthermia resistance, are still poorly understood.

Cirbp belongs to the family of cold shock proteins that are activated under cold stress [10, 59, 61, 71, 73, 137, 138]. Therapeutic hypothermia protected photoreceptors through activating Cirbp pathway [105], while Cirbp exerts neuroprotective effect during therapeutic hypothermia [124]. In addition to cold stress, heat stress also modulates Cirbp expression [14, 82, 87, 130]. In this study, the mRNA and protein expression of Cirbp were significantly down-regulated within a short time after heating treatment, suggesting that Cirbp might act as an acute phase protein in cancer cells under heat stress. A previous study showed that heat treatment also down-regulated Cirbp expression in prostate cancer cells [130]. Moreover, heat-induced Cirbp downregulation was observed in the testes of mice and humans under heat stress condition [14, 82, 87]. These aforementioned findings suggest that Cirbp might be involved in regulating the response of tumor cells to hyperthermia. In this study, our results from in vitro and in vivo experiments demonstrated that Cirbp suppression by RNAi significantly improved the sensitivity of cancer cells and cancer stem-like cells to hyperthermia. On the contrary, exogenous expression of Cirbp almost completely compromised the anti-tumor-killing effect of hyperthermia against cancer cells and cancer stem-like cells in vitro and in vivo, suggesting that ectopic expression of Cirbp induces hyperthermia resistance. Altogether, this work is the first to uncover a previously unrecognized role of Cirbp in regulating hyperthermia resistance and hyperthermic sensitization in cancer.

Of particular interest is how ectopic expression of Cirbp causes hyperthermia resistance and Cirbp silencing sensitizes NPC cells to hyperthermia. Cirbp that is initially described as a DNA damage-induced transcript is a stress-induced protein [73]. Cirbp has been implicated in DNA damage and repair [10, 51, 57, 73], while recent study reported that PARP-1-dependent recruitment of Cirbp promotes double-strand break (DSB) repair and genome stability [10]. Upon hyperthermia-induced DNA damage, tumor cells utilize two primarily distinct kinase signaling cascades to repair DSBs, including the ATM-Chk2 and ATR-Chk1 axes [30, 31, 40, 99, 107, 109, 135]. Moreover, Cirbp plays a crucial role in mediating the associations of MRN and ATM with chromatin [10]. Our results clearly demonstrated that Cirbp knockdown significantly repressed ATM-Chk2 and ATR-Chk1 pathways after hyperthermia, and consequently attenuated DNA damage repair ability of cancer cells and seriously impaired cancer cell survival, ultimately enhancing thermosensitivity in cancer cells and cancer stem-like cells, and tumor growth inhibition (Fig. 12). Conversely, our data showed that during hyperthermia treatment, ectopic expression of Cirbp completely or mostly rescued hyperthermia-induced reduction in the phosphorylation levels of ATM, Chk2, p53, ATR and Chk1, which thereby reversed hyperthermia-induced reduction in DNA damage repair ability of cancer cells and increase in cell apoptosis, ultimately leading to increased thermoresistance and tumor growth, indicating that Cirbp overexpression protects cancer cells against hyperthermia-induced DNA damage and cell death (Fig. 12). Together, these data suggest, for the first time, that exogenous expression of Cirbp induces hyperthermia resistance by promoting DNA damage repair in cancer cells, whereas Cirbp silencing can sensitize cancer cells and cancer stem-like cells to hyperthermic therapy via attenuating the ability of DNA damage repair.

It is well known that CSCs contribute to the resistance to conventional anticancer treatments, such as radiotherapy, chemotherapy, immunotherapy and hyperthermia therapy [3, 7, 8, 25, 29, 43, 52, 76, 77, 81]. The previous studies showed that moderate low temperature preserved the stemness of neural stem cells (NSCs) and prevented cell apoptosis via activation of Cirbp [90], while forced expression of Cirbp under hypoxia could restore the proliferation of NSCs [132], suggesting the importance of Cirbp in the stemness maintenance and self-renewal of stem cells. However, to date, it is still unknown how Cirbp functions in CSCs. Actually, our findings from this study, for the first time, revealed that Cirbp suppression significantly attenuated the stemness of cancer cells, which thereby contributed to noticeably improving the sensitivity of cancer stem-like cells to hyperthermia. On the contrast, this work is the first to reveal that ectopic expression of Cirbp mostly or completely compromised hyperthermia-induced reduction in the stemness of cancer cells, which thereby contributed to the resistance of cancer stem-like cells to hyperthermia. It is clear that our above findings are in line with the functions of Cirbp on stemness in NSCs [90, 132]. Since CSCs are responsible for the resistance to anticancer therapy, such as hyperthermia therapy [3, 7, 8, 25, 29, 43, 52, 76, 77, 81], the functions of Cirbp in the stemness maintenance of CSCs also contributes to the hyperthermia resistance or hyperthermic radiosensitization in our study.

It is well known that a single miRNA can regulate a large number of target protein-coding genes involved in different signal transduction pathways that participate in many physiological and pathological processes, including tumor formation and progression [9, 20, 28, 80, 91, 133]. Therefore, miRNAs are being considered as potential therapeutic targets for various diseases, including hepatitis and cancers [9, 20, 28, 80, 91, 133]. Several miRNA mimics and molecules that target miRNAs (anti-miRs) have shown promise for clinical application in preclinical or clinical trials [65, 89], and miRNA-targeted therapeutics have already been tested in clinical trials, including a mimic of the tumor suppressor miR-34, which reached phase I clinical trials for cancer treatment [65] and anti-miRs targeting miR-122, which reached phase II trials for hepatitis treatment [42]. More importantly, in vitro chemical synthesis and in vivo delivery of miRNAs for cancer therapy is very handy [9, 20, 28, 80, 91, 133]. In addition, the previous study indicated that some miRNAs (i.e., miR-142-5p and miR-143) were identified to belong to temperature-sensitive miRNAs (termed thermomiRs) [123]. In this study, Cirbp is identified to be a direct target of thermomiR-377-3p in NPC cells, while thermomiR-377-3p improves the sensitivity of NPC cells and cancer stem-like cells to hyperthermia by directly suppressing Cirbp expression, suggesting that thermomiR-377-3p is a promising therapeutic targets of hyperthermia for NPC.

The use of hyperthermia as a treatment for cancer is not new and dates back to the work of Coley [18, 129], which has a wide variety of biological effects. In recent years, a large number of in vitro and in vivo experiments and clinical data demonstrate that as an adjunctive therapy, hyperthermia combined with radiotherapy and/or chemotherapy improves clinical outcome in cancer therapy [2, 23, 45, 50]. More specifically, hyperthermia has been confirmed to improve response to chemoradiation therapy in patients with soft tissue sarcoma [60], liver cancer [103] and comprehensively raise therapeutic effect to radiation in several clinical trials in patients who have head and neck [21, 110], melanoma [83], breast [44, 66, 113], advanced cervical [32, 111, 139] and brain cancer [27, 48, 100]. Currently, the standard therapy for patients with NPC is radiotherapy combined with chemotherapy [13, 17, 106]. In contrast to other solid cancers [2, 6, 22, 23, 29, 45, 50, 86, 101, 119], the hyperthermia is relatively less investigated in NPC. In the field of NPC, a small amount of clinical trials preliminarily demonstrated that hyperthermia combined with radiation therapy can improve progression-free survival and local progression-free survival of NPC patients, although no increase in overall survival was observed [37, 46, 85, 121]. In this study, our findings firstly revealed that hyperthermia dramatically attenuated the stemness of NPC cells, while combination treatment of hyperthermia and Ori significantly increased the anti-tumor killing effect on NPC cells and CSC-like population within NPC cells. Moreover, our results also indicated that hyperthermia substantially improved the sensitivity of radiation-resistant NPC cells and CSC-like cells to radiotherapy. Collectively, this work is first to uncover that hyperthermia alone or combined with radiotherapy or chemotherapy can effectively eliminate CSC-like population within NPC cells.

Natural product Ori is the major active ingredient of the traditional Chinese medicinal herb Rabdosia rubescens, and has anti-tumor activity [38, 55, 72, 74, 78, 84, 94, 102, 112, 122, 126–128, 134, 136] and anti-inflammatory [34]. Ori and its analogue alone or combined with chemotherapy and radiotherapy was reported to effectively kill tumor cells of leukemia, ovarian cancer, lung cancer, esophageal squamous cell carcinoma, osteosarcoma, breast cancer, colorectal cancer and prostate cancer [38, 55, 72, 74, 78, 84, 94, 102, 112, 122, 126–128, 134, 136]. As CSCs have been identified as the main center of cancer therapeutic resistance [26, 39, 53], eradicating CSCs is considered as an effective and powerful strategy to improve current anti-cancer therapeutics [26, 39, 53]. However, it remains unknown whether Ori and its analogue alone or combined with hyperthermia can effectively kill CSCs. Our work is the first to reveal that Ori treatment alone or combined with hyperthermia effectively eliminates CSC-like population within cancer cells, suggesting that Ori is active against cancer stem-like cells, but the targets of Ori remain to be fully investigated.

## Conclusions

In summary, this work is the first to identify a previously unrecognized mechanism of hyperthermia resistance that Cirbp causes hyperthermia resistance by enhancing DNA damage repair in cancer. ThermomiR-377-3p and its target gene Cirbp are important regulators of thermosensitivity and may represent important targets of hyperthermia for further development.

### Supplementary Information

**Supplementary Material 1.**

**Supplementary Material 2.**

## Data Availability

All data generated or analyzed during the current study are included in this published article (and its supplementary information files).
