# Supplementary material for: ThermomiR-377-3p-induced suppression of Cirbp expression is required for effective elimination of cancer cells and cancer stem-like cells by hyperthermia
Source: J Exp Clin Cancer Res. 2024 Feb 29;43:62. doi: 10.1186/s13046-024-02983-3 (PMC10903011; doi:10.1186/s13046-024-02983-3)
Supplement: Supplementary file 1 — Supplementary Material 1. [file 13046_2024_2983_MOESM1_ESM.pdf]

Table S1 Primers for qRT-PCR, oligonucleotide sequence and miRNA sequence

Name	Sequences (5'-3')
**Primers for qRT-PCR**	
GAPDH-F	ACCCAGAAGACTGTGGATGG
GAPDH-R	TCTAGACGGCAGGTCAGGTC
ABCG2-F	AGCAGCTCTTCGGCTTGCAACA
ABCG2-R	GTTCCAACCTTGGAGTCTGCCACT
Oct4-F	CTTGCTGCAGAAGTGGGTGGAGGAA
Oct4-R	CTGCAGTGTGGGTTTCGGGCA
Bmi-1-F	CGTGTATTGTTCGTTACCTGGA
Bmi-1-R	TTCAGTAGTGGTCTGGTCTTGT
Nanog-F	AAGGTCCCGGTCAAGAAACAG
Nanog-R	CTTCTGCGTCACACCATTGC
Sox2-F	GCCGAGTGGAAACTTTTGTCG
Sox2-R	GGCAGCGTGTACTTATCCTTCT
Cirbp-F	AGGGCTGAGTTTTGACACCAA
Cirbp-R	ACAAACCCAAATCCCCGAGAT
RBM3-F	TGGGAGGGCTCAACTTTAAC
RBM3-R	ATGCTCTGGGTTGGTGAAG
Bcl-2-F	AGGCTGGGATGCCTTTGTGGAA
Bcl-2-R	CAAGCTCCCACCAGGGCCAAA
BCL2L1-F	GGAGAACGGCGGCTGGGATA
BCL2L1-R	GGCCACAGTCATGCCCGTCA
BIRC3-F	GATGTTTCAGATCTACCAGTG
BIRC3-R	GAAATGTACGAACTGTACCCT
BIRC4-F	GTCAGAACACAGGCGACACTT
BIRC4-R	ATCACCTTCACCTAAAGCATA
BIRC5-F	CTCAAGGACCACCGCATC
BIRC5-R	ATCTGCAAGGGACAGCACAG
Fas-F	GGACCCTCCTACCTCTGGTT
Fas-R	ACCTGGAGGACAGGGCTTAT
Bad-F	CGAGATCGGGCTTGGGGTGAG
Bad-R	CTGGGCCCTCATCTGTCTGCC
CRADD-F	AGTACTCCGCTCACTTCGC
CRADD-R	CTGCAGGCAGGTCGGTCAT

Bid-F	GGGTAGTCGACCGTGTCCGC
Bid-R	GCTGGAACCGTTGTTGACCTCAC
Bik-F	TTTGGAATGCATGGAGGGCA
Bik-R	TCTAAGAACATCCCTGATGT

miRNA primers for qRT-PCR

U6 snRNA stem-loop	GAATTTGCGTGTCATCCTTGC
U6 snRNA-F	CTCGCTTCGGCAGCACATA
U6 snRNA-R	GAATTTGCGTGTCATCCTTGC
miR-377-3p stem-loop	CTCAACTGGTGTCGTGGAGTCGGCAATTCAGTTGAGACAAAAGT
miR-377-3p-F	ACACTCCAGCTGGGATCACACAAAGGCAAC
miR-377-3p-R	TGGTGTCGTGGAGTCG
miR-381-3p stem-loop	CTCAACTGGTGTCGTGGAGTCGGCAATTCAGTTGAGACAGAGAGAG
miR-381-3p-F	ACACTCCAGCTGGGTATACAAGGGCAAGCT
miR-381-3p-R	TGGTGTCGTGGAGTCG
miR-142-5p stem-loop	CTCAACTGGTGTCGTGGAGTCGGCAATTCAGTTGAGAGTAGTGC
miR-142-5p-F	ACACTCCAGCTGGGCATAAAGTAGAAAGC
miR-142-5p-R	TGGTGTCGTGGAGTCG
miR-143 stem-loop	CTCAACTGGTGTCGTGGAGTCGGCAATTCAGTTGAGGAGCTACA
miR-143-F	ACACTCCAGCTGGGTGAGATGAAGCACTG
miR-143-R	TGGTGTCGTGGAGTCG
miR-124-3p stem-loop	CTCAACTGGTGTCGTGGAGTCGGCAATTCAGTTGAGGGCATTCA
miR-124-3p-F	ACACTCCAGCTGGGTAAGGCACGCGGTG
miR-124-3p-R	TGGTGTCGTGGAGTCG
miR-145-5p stem-loop	CTCAACTGGTGTCGTGGAGTCGGCAATTCAGTTGAGAGGGATTC
miR-145-5p-F	ACACTCCAGCTGGGGTCCAGTTTTCCCAGGA
miR-145-5p-R	TGGTGTCGTGGAGTCG
miR-27a-3p stem-loop	CTCAACTGGTGTCGTGGAGTCGGCAATTCAGTTGAGGCGGAACT
miR-27a-3p-F	ACACTCCAGCTGGGTTCACAGTGGCTAAG
miR-27a-3p-R	TGGTGTCGTGGAGTCG
miR-27b-3p stem-loop	CTCAACTGGTGTCGTGGAGTCGGCAATTCAGTTGAGGCAGAACT
miR-27b-3p-F	ACACTCCAGCTGGGTTCACAGTGGCTAAG
miR-27b-3p-R	TGGTGTCGTGGAGTCG
miR-300 stem-loop	CTCAACTGGTGTCGTGGAGTCGGCAATTCAGTTGAGAGAGAGAG
miR-300-F	ACACTCCAGCTGGGTATACAAGGGCAGACT
miR-300-R	TGGTGTCGTGGAGTCG

Oligonucleotide sequence used for RNAi in this study

shRNA-NC	CGTGATCTTCACCGACAAGAT
shCirbp-1	CGACAGTTACGCTACACACAA
shCirbp-2	CTTCTCAAAGTACGGACAGAT
shCirbp-3	GCCATGAATGGGAAGTCTGTA
shCirbp-4	CGGGTCCTACAGAGACAGTTA
**miRNAs sequence for miR-377-3p and miR-381-3p**	
mimics-NC	UUCUCCGAACGUGUCACGUTT
miR-377-3p mimics	AUCACACAAAGGCAACUUUUGU
miR-381-3p mimics	UAUACAAGGGCAAGCUCUCUGU
inhibitor-NC	CAGUACUUUUGUGUAGUACAA
miR-377-3p inhibitor	ACAAAAGUUGCCUUUGUGUGAU
miR-381-3p inhibitor	ACAGAGAGCUUGCCCUUGUAUA

Table S2 List of antibodies and suppliers used for immunoblotting and immunofluorescence (IF)

Antibody	Cat. No	Company	Mol. Weight	Dilution
GAPDH	10494-1-AP	Proteintech	36 kDa	1:10000 for WB
Cirbp	10209-2-AP	Proteintech	19 kDa	1:1000 for WB
ABCG2	10051-1-AP	Proteintech	70 kDa	1:1000 for WB
Nanog	Ab109250	Abcam	35 kDa	1:1000 for WB
Sox2	Ab97959	Abcam	34 kDa	1:1000 for WB
Oct4	Ab200834	Abcam	38 kDa	1:1000 for WB
Bmi-1	6964	CST	42 kDa	1:1000 for WB
E-cadherin	610181	BD Biosciences	120 kDa	1:250 for WB
Vimentin	550513	BD Biosciences	57 kDa	1:500 for WB
N-cadherin	610920	BD Biosciences	130 kDa	1:250 for WB
β-catenin	8480	CST	92 kDa	1:1000 for WB
p-p53	9286S	CST	53 kDa	1:1000 for WB
p-BRCA1	9009S	CST	220 kDa	1:1000 for WB
ATM	2873S	CST	350 kDa	1:1000 for WB
p-ATM	13050S	CST	350 kDa	1:1000 for WB
ATR	13934S	CST	300 kDa	1:1000 for WB
p-ATR	2853S	CST	300 kDa	1:1000 for WB
Chk1	2360S	CST	56 kDa	1:1000 for WB
p-Chk1	2348S	CST	56 kDa	1:1000 for WB
Chk2	3440S	CST	62 kDa	1:1000 for WB
p-Chk2	2197S	CST	62 kDa	1:1000 for WB
γ-H2AX	Ab26350	Abcam	16 kDa	1:1000 for WB<br>1:500 for IF
53BP1	Ab175933	Abcam	450 kDa	1:1000 for WB<br>1:250 for IF

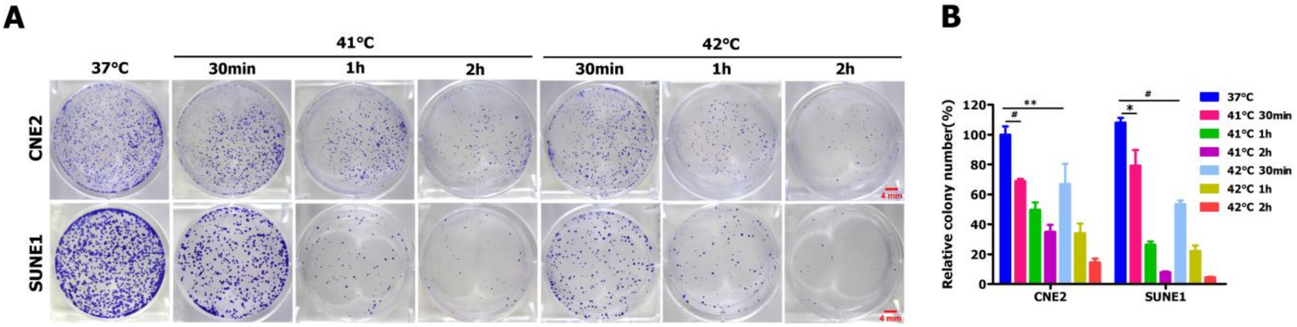

Figure S1 Effects of hyperthermia treatment on NPC cell proliferation were measured by colony formation assay.

Colony formation assay was performed to in vitro test the proliferation ability of CNE2 and SUNE1 cells treated with hyperthermia at 41°C or 42°C for 30min, 1h or 2h. Representative pictures of colony formation assay of the indicated cancer cells were presented in Figure S1A, while the quantification of colony formation of the indicated cancer cells were provided in Figure S1B.

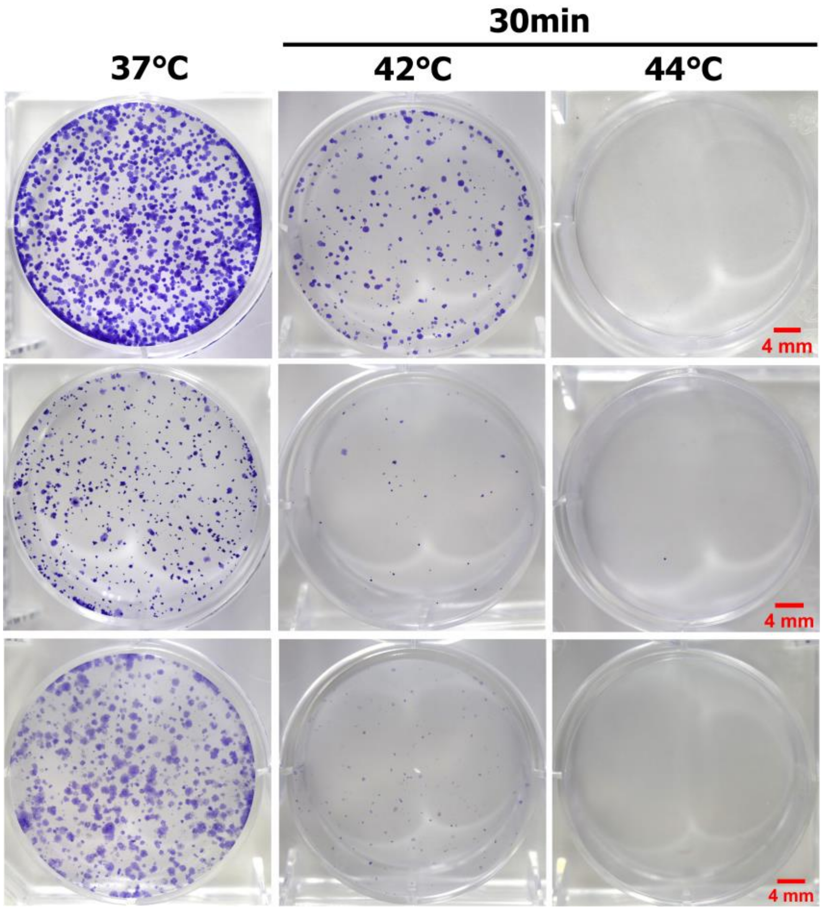

Figure S2 Effects of hyperthermia treatment on NPC cell proliferation were measured by colony formation assay.

Colony formation assay was performed to in vitro test the proliferation ability of CNE2, SUNE1 and HONE1-EBV cells treated with hyperthermia at 42°C or 44°C for 30min. Representative pictures of colony formation assay of the indicated cancer cells were here presented in Figure S2, while the statistical data on the proliferation rate of the indicated cancer cells were provided in Figure 1B.

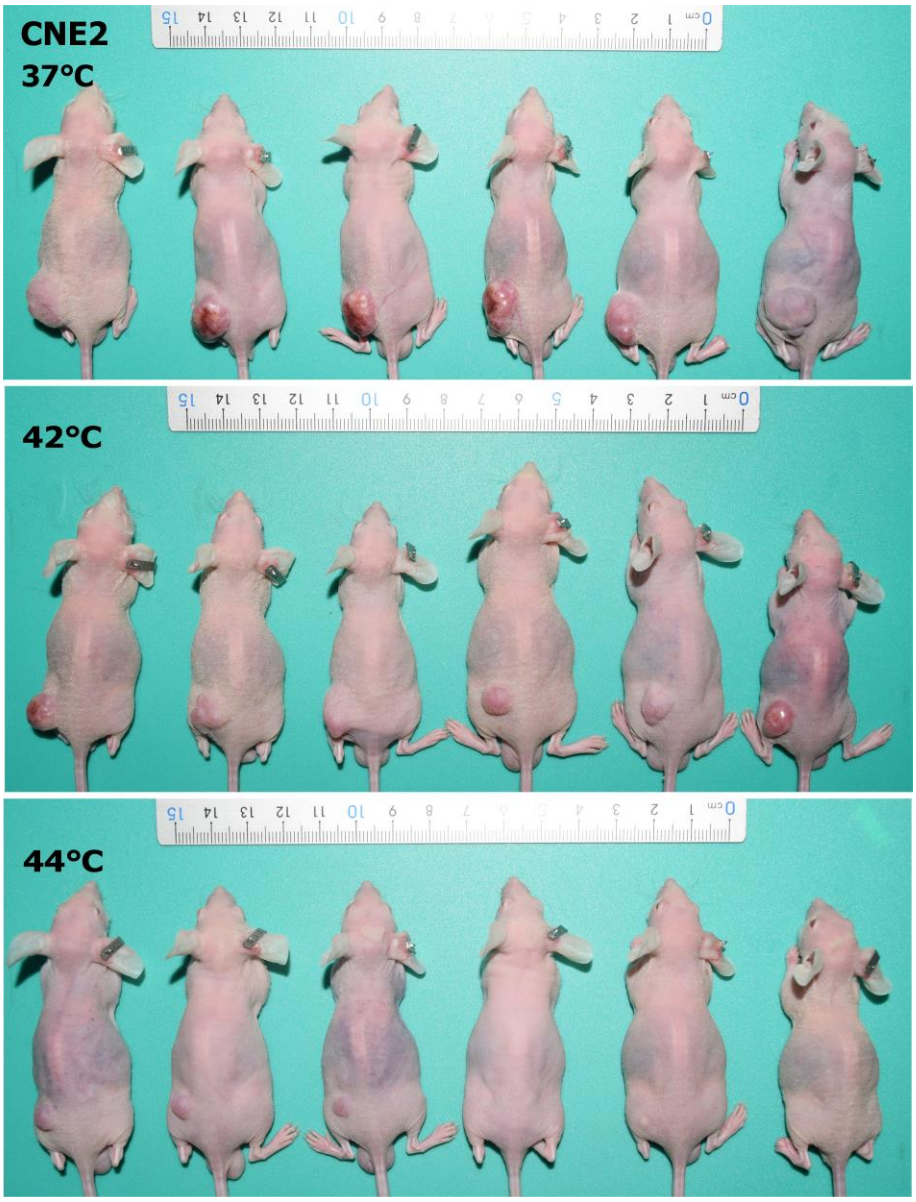

Figure S3 The xenograft subcutaneous tumor formation of hyperthermia-treated CNE2 cells in nude mice.

As mentioned in Materials and Methods section, CNE2 cells in vitro treated with hyperthermia at 42°C or 44°C for 30min were injected subcutaneously into nude mice (n=6). Tumor size was estimated by serial calipation. Representative pictures of nude mice harbouring tumor xenografts at the end of experiment were presented in Figure S3, while representative images of stripped tumor xenografts at the end of experiment, growth curve of tumor volumes and tumor weight were provided in Figure 1C-E, respectively.

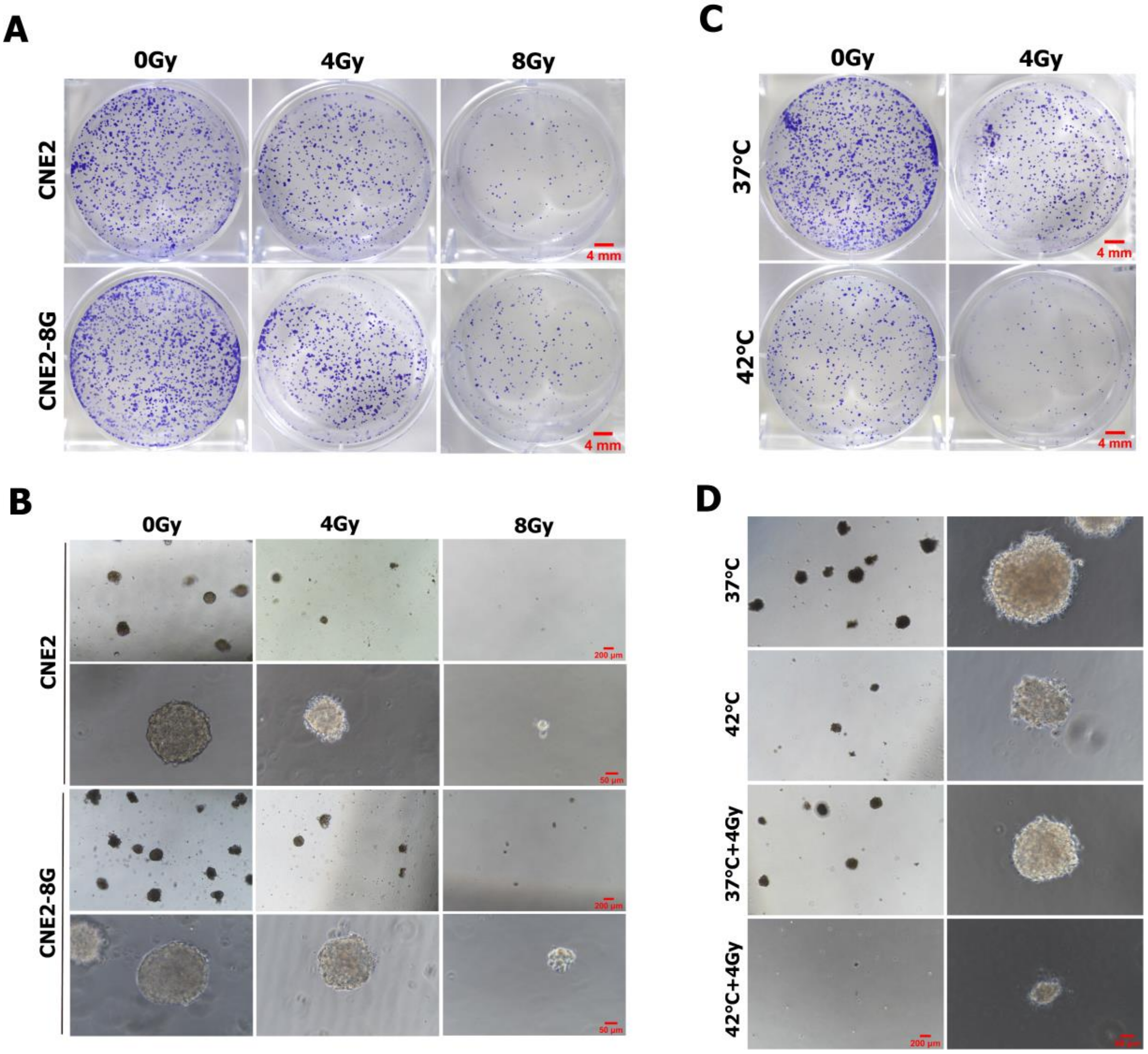

Figure S4 Hyperthermia treatment increased radiation sensitivity of NPC cells resistant to radiation.

(**A-B**) Representative photographs of colony formation (A) and tumor sphere formation (B) assays performed in CNE2 and CNE2-8G cells exposed to irradiation (IR) treatment at 0, 4 and 8 Gy.

The quantification of colonies and tumor spheres is shown in Figure 3G and Figure 3H, respectively.

(**C-D**) Representative photographs of colony formation (C) and tumor sphere formation (D) assays which were performed in CNE2-8G cells treated by hyperthermia (42°C for 30min) and IR (4 Gy) alone or combined.

The quantification of colonies and tumor spheres is shown in Figure 3J and Figure 3K, respectively.

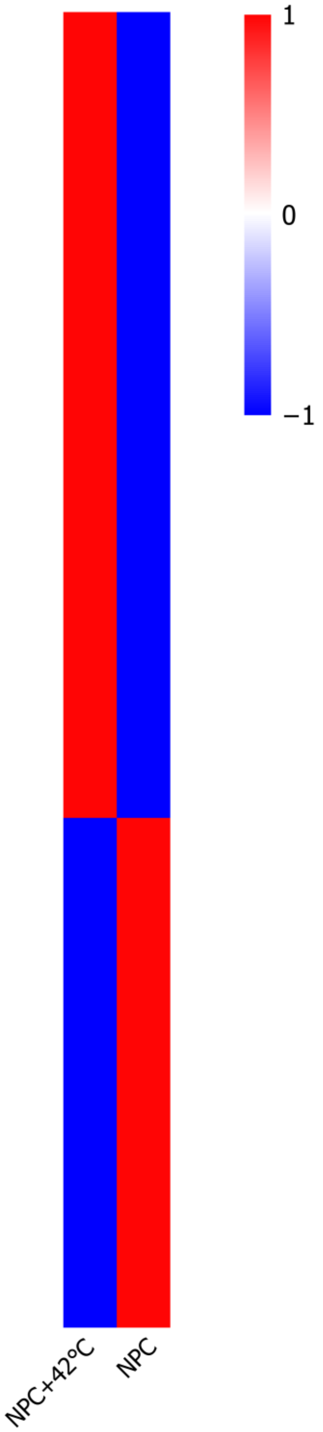

Figure S5 Class comparison and hierarchical clustering of differentially expressed genes between NPC cells treated with or without hyperthermia at 42°C for 30min.

A cluster heat map for differentially expressed genes (see Table S3) is shown.

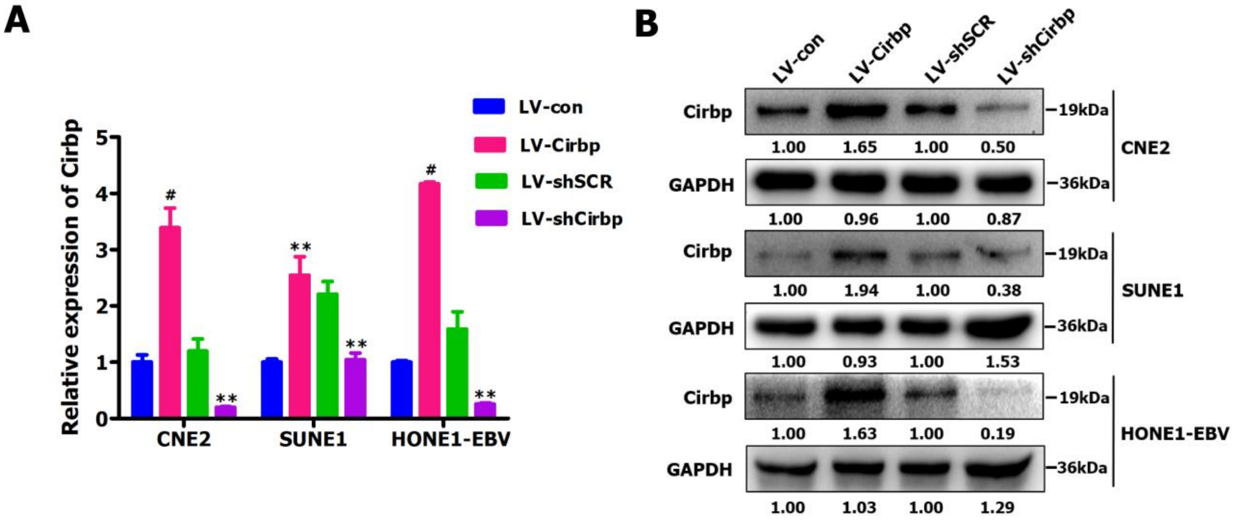

Figure S6 NPC cells were successfully transduced by lentiviral vectors carrying Cirbp or shCirbp.

qRT-PCR (A) and Western blot (B) analysis of Cirbp expression in the NPC cells transduced with lentivirus carrying Cirbp or shCirbp.

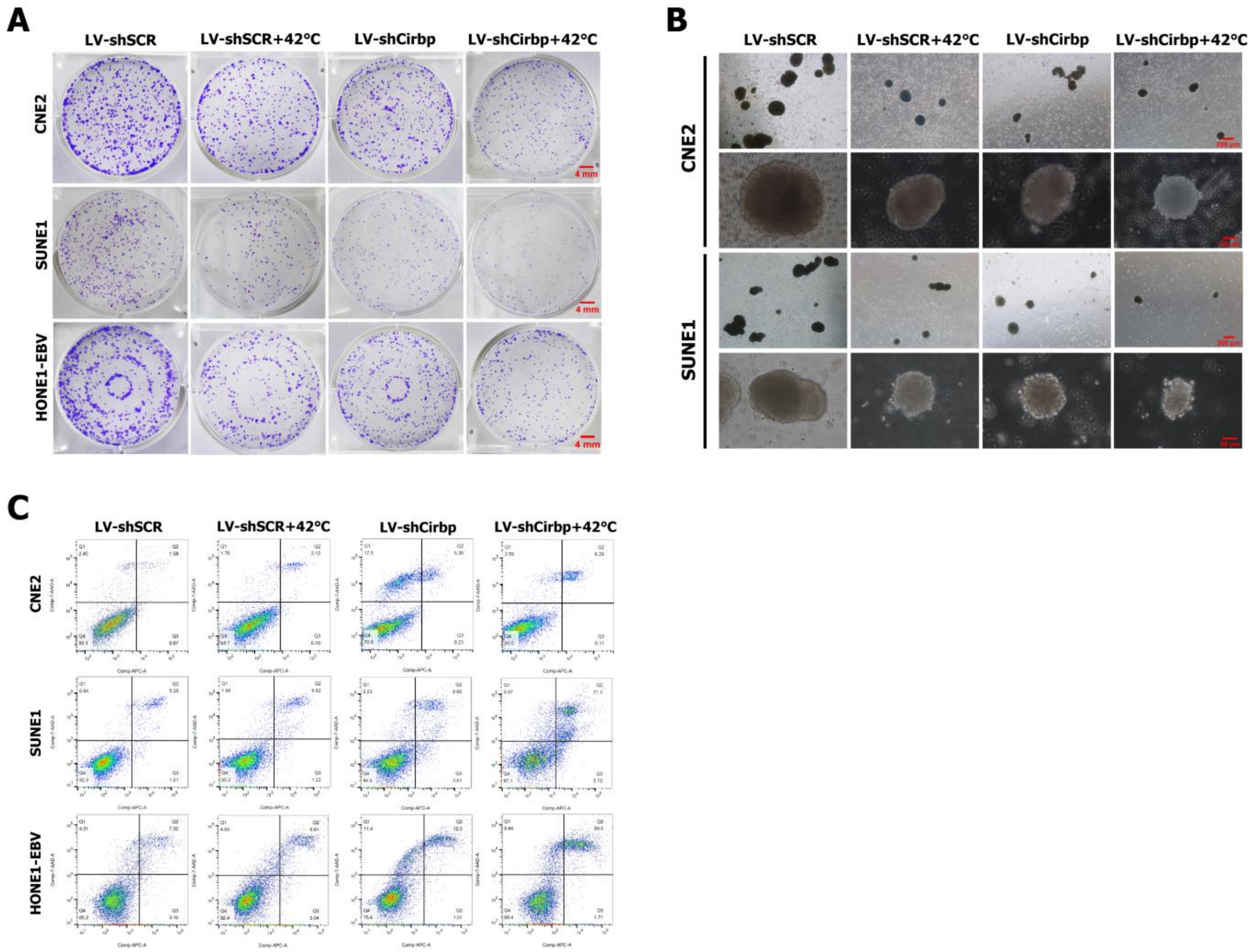

Figure S7 RNAi-mediated silencing of endogenous Cirbp enhanced the killing effect of hyperthermia on NPC cells in vitro.

Colony formation assay (A), tumor sphere formation assay (B) and AnnexinV/PI apoptosis assay (C) were performed in shSCR- or shCirbp-expressing NPC cells treated with or without hyperthermia at 42°C for 30min. Representative images of colony formation assay (A), tumor sphere formation assay (B) and AnnexinV/PI apoptosis assay (C) were here presented in Figure S7, while the statistical data on colony formation assay, tumor sphere formation assay and AnnexinV/PI apoptosis assay were provided in Figure 4F, Figure 4G and Figure 4H, respectively.

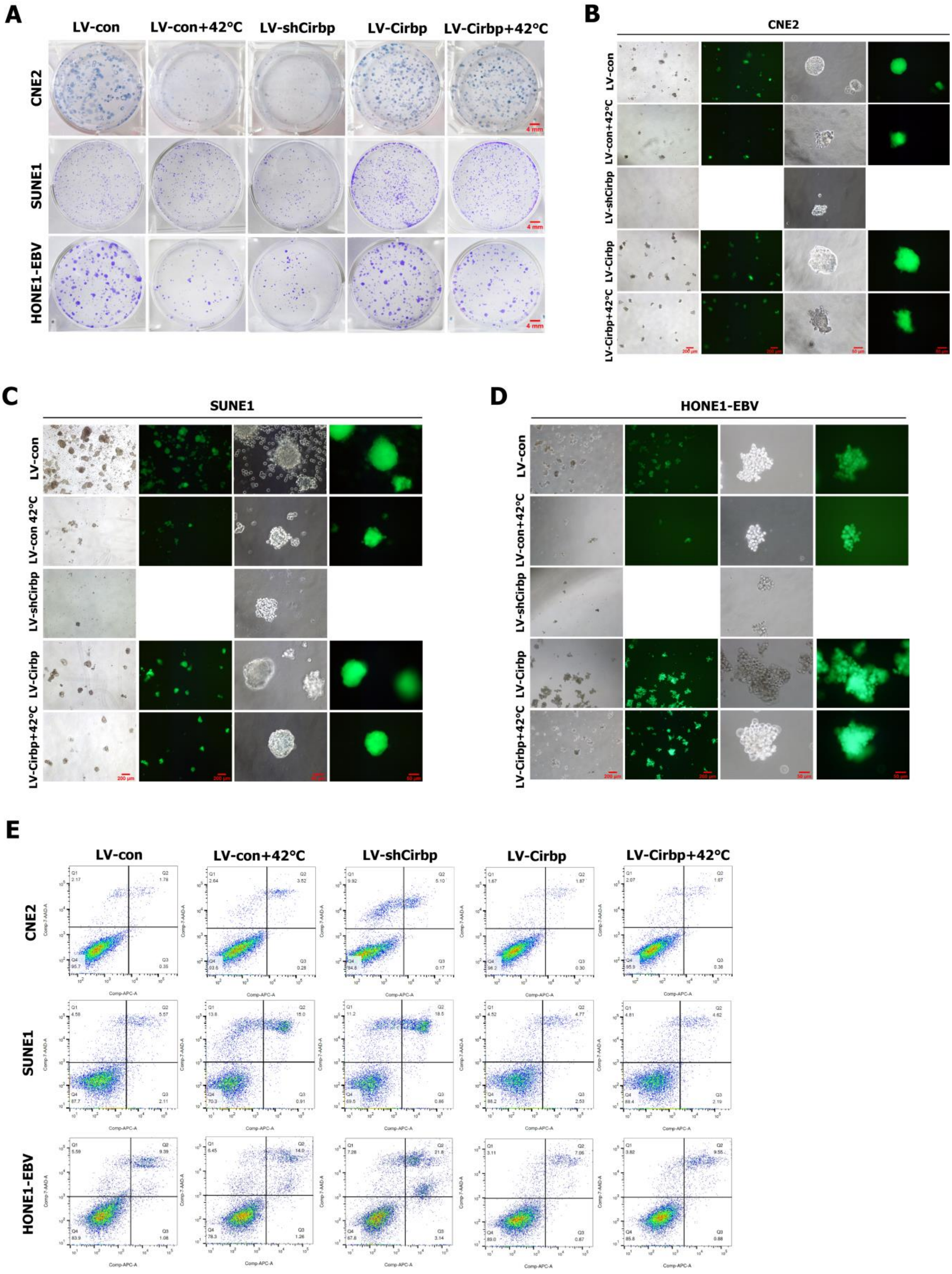

Figure S8 Exogenous expression of Cirbp counteracted the killing effect of hyperthermia on NPC cells in vitro.

Colony formation assay (A), tumor sphere formation assay (B-D) and AnnexinV/PI apoptosis

assay (E) were performed in Cirbp-expressing NPC cells treated with or without hyperthermia at 42°C for 30min. Representative images of colony formation assay (A), tumor sphere formation assay (B-D) and AnnexinV/PI apoptosis assay (E) were here presented in Figure S8, while the statistical data on colony formation assay, tumor sphere formation assay and AnnexinV/PI apoptosis assay were provided in Figure 5B, Figure 5C and Figure 5D, respectively.

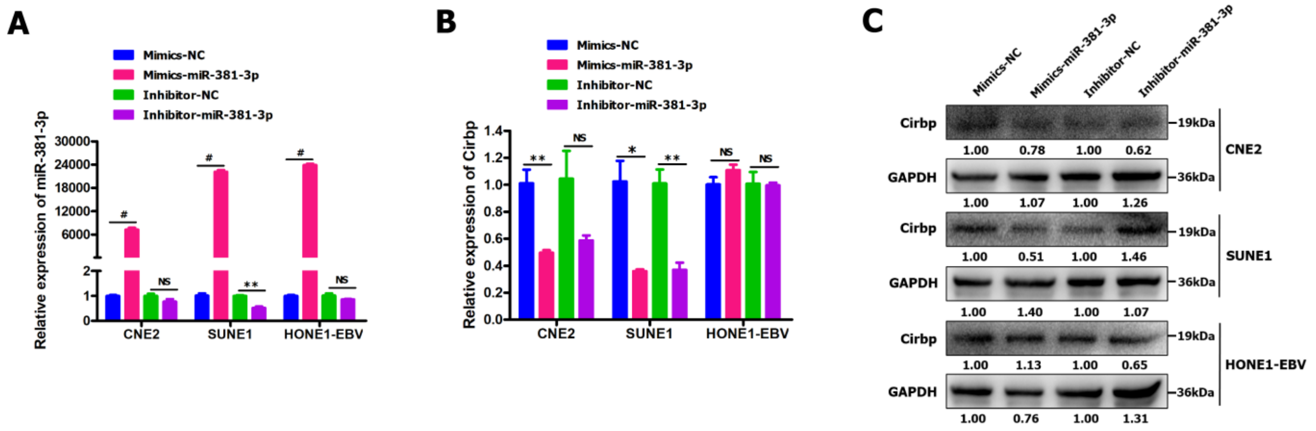

Figure S9 Detection of the expression of miR-381 (A) and Cirbp (B and C) in NPC cells transiently transfected with miR-381-3p mimics or inhibitor by qRT-PCR (A and B) and Western blotting (C).

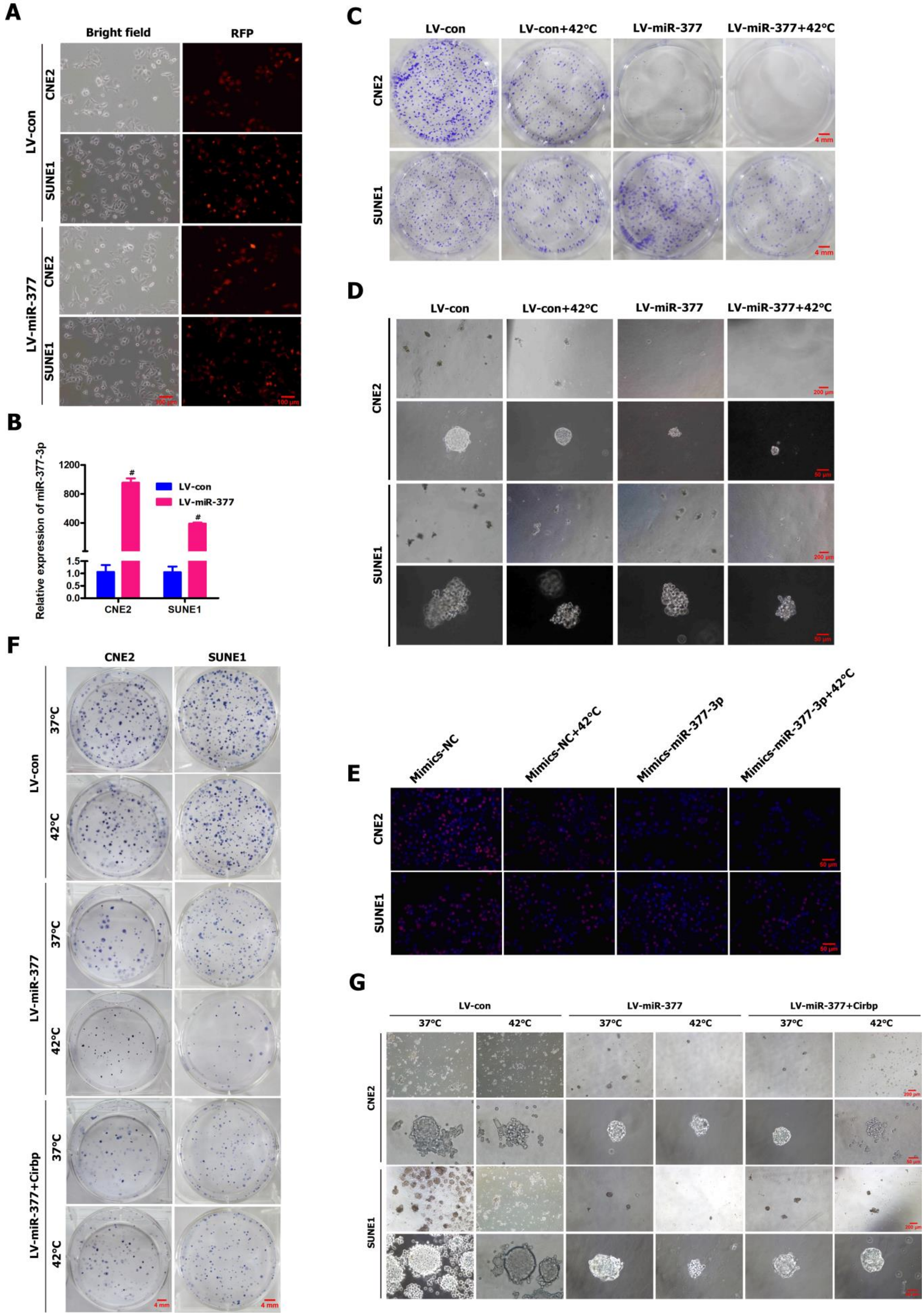

Figure S10 ThermomiR miR-377-3p improved the sensitivity of NPC cells and

cancer stem-like cells to hyperthermia *in vitro* by directly suppressing Cirbp expression.

(**A**) NPC cells were successfully infected by lentivirus carrying miR-377 and RFP.

(**B**) qRT-PCR analysis of miR-377-3p expression in the NPC cells infected with lentivirus carrying miR-377 and RFP.

(**C**) Colony formation assay was performed in miR-377-expressing NPC cells treated with or without hyperthermia at 42°C for 30min.

Representative images of colony formation assay were here presented in Figure S10C, while the statistical data on colony formation assay were provided in Figure 6I.

(**D**) Tumor sphere formation assay was performed in miR-377-expressing NPC cells treated without or with hyperthermia at 42°C for 30min.

Representative images of tumor sphere formation assay were here presented in Figure S10D, while the statistical data on tumor sphere formation assay were provided in Figure 6J.

(**E**) EdU assay was performed in NPC cells transiently transfected with miR-377-3p mimics, and then treated with or without hyperthermia at 42°C for 30min.

Representative images of EdU assay were here presented in Figure S10E, while the statistical data on EdU assay were provided in Figure 6K.

(**F-G**) Colony formation assay (F) and tumor sphere formation assay (G) were performed in miR-377- and Cirbp-expressing NPC cells treated with or without hyperthermia at 42°C for 30min.

Representative images of colony formation assay (F) and tumor sphere formation assay (G) were here presented in Figure S10, while the statistical data on colony formation assay and tumor sphere formation assay were provided in Figure 6M and Figure 6N, respectively.

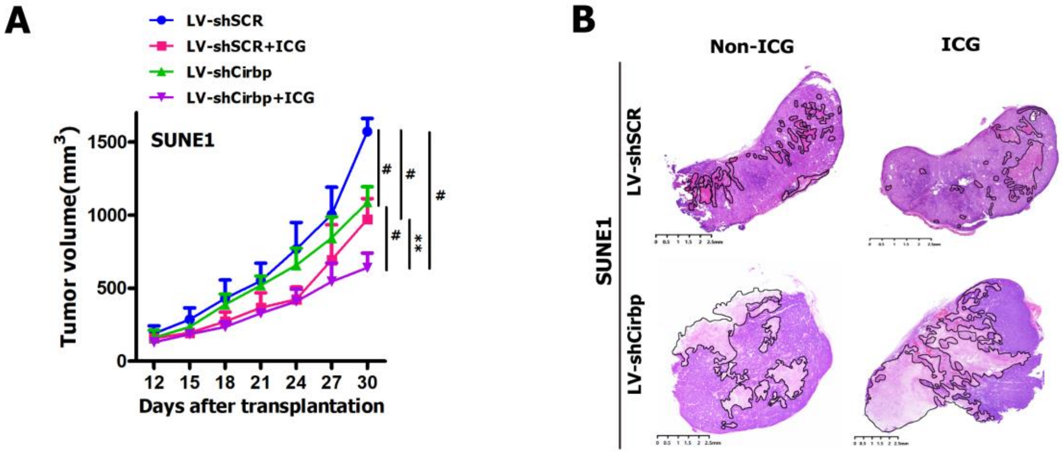

Figure S11 RNAi-mediated silencing of endogenous Cirbp promoted the killing effect of hyperthermia on NPC cells in vivo.

(**A**) The tumor growth curve (n=4 mice/group).

(**B**) Representative pictures of H&E staining of stripped xenograft tumors.

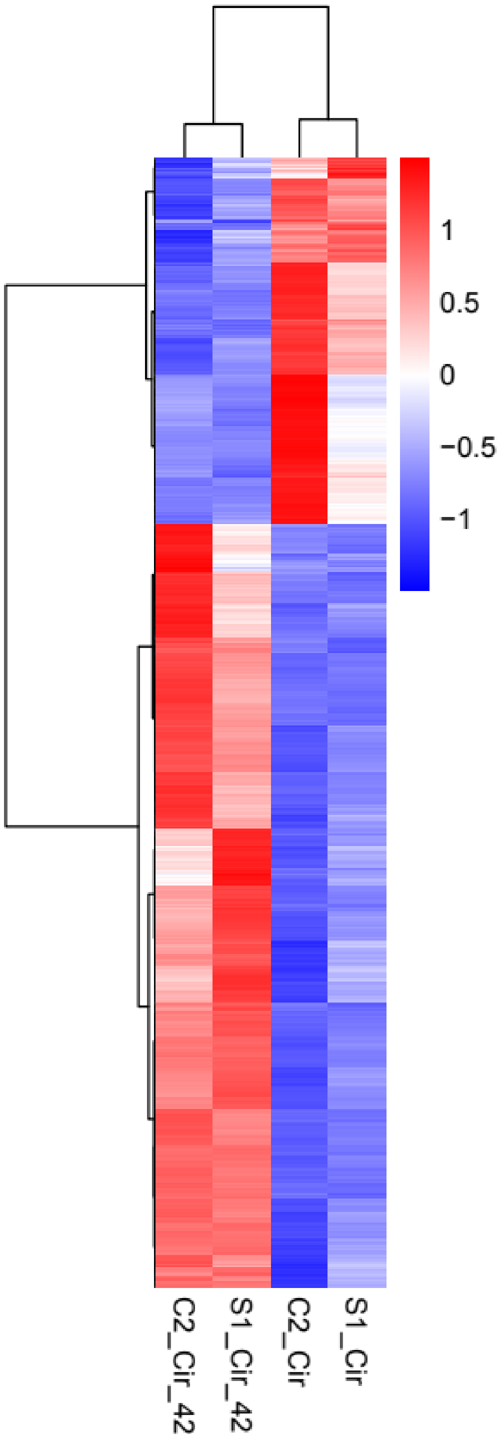

Figure S12 Class comparison and hierarchical clustering of differentially expressed genes between Cirbp-expressing NPC cells plus 42 ℃ and Cirbp-expressing NPC cells.

A cluster heat map for differentially expressed genes (see Table S9) is shown. C2: CNE2; S1：SUNE1; Cir: Cirbp.
